# Four methods for estimating hepatitis C incidence using extant testing data

**DOI:** 10.1371/journal.pone.0335115

**Published:** 2026-06-10

**Authors:** William J. McFarlane, Jennifer A. Flemming, Susan B. Brogly, Yingwei Peng

**Affiliations:** 1 Department of Public Health Sciences, Queen’s University, Kingston, Ontario, Canada; 2 ICES Queen’s, Kingston, Ontario, Canada; 3 Department of Medicine, Queen’s University, Kingston, Ontario, Canada; 4 Department of Surgery, Queen’s University, Kingston, Ontario, Canada; Kerman University of Medical Sciences, IRAN, ISLAMIC REPUBLIC OF

## Abstract

**Background:**

Accurate estimation of hepatitis C (HCV) incidence is crucial for measuring progress towards HCV elimination targets set by the World Health Organization (WHO). Extant HCV antibody (Ab) and RNA test results are widely used to estimate HCV incidence, but the impact of cohort specification and case definition on validity and generalizability is poorly understood.

**Methods:**

Using databases linked at ICES – a repository of administrative health data for Ontario residents – we constructed a cohort of 15.8 million Ontarians aged 18–80 between 1999 and 2018 to estimate annual HCV incidence using four methods. The population-based method calculated HCV incidence as the number of new HCV diagnoses each year divided by annual population size estimates, while Poisson regression was used in the other three incidence estimation methods: the test-negative method defined eligibility at first negative test; the RNA-based method prioritized specificity by requiring RNA+ tests; and the antibody-inclusive method prioritized sensitivity by including all Ab+ tests.

**Results:**

Distinct patterns of HCV incidence were found across the estimation methods: the RNA-based estimates were lowest and fluctuated around 30 cases per 100,000 person-years, while population-based and antibody-inclusive estimates were 1.5-fold higher, and test-negative estimates were 7.9-fold higher. Population-based estimates were sensitive to changes in the HCV case definition used in Ontario from 1999–2018. The test-negative cohort had a high prevalence of human immunodeficiency virus (HIV) and substance use disorder, limiting generalizability of HCV incidence estimates. RNA-based estimates likely underestimated HCV incidence because 22% of Ab+ tests were unconfirmed by RNA testing, while antibody-inclusive estimates likely overestimated HCV incidence by assuming all unconfirmed Ab+ tests were true cases.

**Conclusion:**

These new findings illustrate the influence of cohort definition and HCV case definition when estimating HCV incidence using extant testing data, which will support accurate measurement of progress towards WHO HCV elimination goals.

## Introduction

Approximately 242,000 individuals died from hepatitis C (HCV) infection globally in 2022 and around one million new HCV cases occur each year [1]. Updated global HCV elimination targets were announced by the World Health Organization (WHO) in 2023, which included a reduction in HCV incidence to ≤5 new annual HCV cases per 100,000 individuals [2]. Since the quantity and quality of HCV testing data varies across regions, several approaches have been proposed for measuring progress towards this HCV incidence target [2–5].

Achievement of HCV incidence targets would ideally be measured by prospectively testing for HCV in a representative cohort of adults that were HCV negative (HCV-) at baseline, but the required resources make this approach infeasible in many regions [3]. Alternatively, extant HCV antibody (HCV Ab) and HCV ribonucleic acid (RNA) test results conducted in public laboratories are used to estimate HCV incidence in many regions, including Australia, Canada, and the USA [6–8]. This data source is efficient but does not clearly differentiate prevalent and incident HCV infections, so the potential for misclassification of HCV status is high. There are different approaches for defining HCV susceptibility and incident HCV infection when using extant testing data, and the impact of these methodological decisions on misclassification and generalizability is poorly understood.

The simplest way to use extant testing data to estimate HCV incidence is a ratio of the number of reported HCV infections to the estimated population size. Issues with this method include changes in HCV case definitions over time, and difficulty differentiating incident and prevalent HCV infections [9]. Chronic HCV infection can persist for decades without symptoms, making it difficult to determine when infection occurred [10]. Incident and prevalent infections are better differentiated using a cohort that defines eligibility based on first testing HCV- [11,12]. Generalizability of HCV incidence estimates may be limited when using this approach because HCV screening is risk-based in Ontario; test-negative cohorts therefore tend to favor patients with a high risk of HCV such as individuals infected with human immunodeficiency virus (HIV) or with a history of injection drug use (IDU) [13,14].

HCV incidence can also be estimated using a sample of the general population, by considering individuals HCV- if they have no history of an HCV positive (HCV+) test result. This design improves generalizability compared to the test-negative method, but is more likely to misclassify prevalent HCV infections as incident. One way to increase the specificity of HCV classification is to only consider positive HCV RNA tests (RNA+) when defining incident HCV infection. The HCV testing cascade begins with an HCV Ab test (measuring lifetime HCV exposure); those testing HCV Ab positive (Ab+) should then receive a confirmatory HCV RNA test (measuring current/active HCV infection) [13]. However, up to 44% of individuals that test HCV Ab + do not receive a confirmatory HCV RNA test (i.e., an unconfirmed HCV Ab+ result) [15]. HCV RNA testing often requires an additional blood draw following an HCV Ab+ test result, and unconfirmed HCV Ab+ tests may occur because patients don’t attend RNA testing appointments or are unavailable for scheduling [13].

It is possible that many individuals with unconfirmed Ab+ test results were currently/actively infected with HCV at the time they tested Ab+, because 75–85% of untreated HCV infections become chronic and RNA testing is a prerequisite for HCV treatment [10,13,16]. Limiting the definition of HCV infection to RNA+ test results would therefore prioritize specificity, but would likely underestimate HCV incidence because some of the unconfirmed Ab+ tests came from individuals who would have tested HCV RNA+ (if tested). Conversely, sensitivity can be maximized by defining incident HCV infection as an RNA+ test or an individual’s first recorded Ab+ test result. This would include all unconfirmed Ab+ tests as cases, likely overestimating HCV incidence because not all of these individuals would have tested HCV RNA+ (if tested).

Extant HCV testing data are an efficient data source for estimating HCV incidence, with data repositories for many regions including the integrated Public Health Information System for Ontario and the National Notifiable Diseases Surveillance System for the USA [17,18]. When prospective cohort studies are infeasible, regions often rely on extant HCV testing data to measure progress towards WHO HCV elimination targets [2,3,5]. HCV incidence can be measured using extant HCV testing data through a variety of approaches, the strengths and limitations of which are poorly understood. Therefore, the objective of this study was to use extant HCV testing data to estimate the annual incidence of HCV from 1999 to 2018 in Ontario using four different methods, and comment on the generalizability and potential biases for each method.

## Methods

### Data sources

The study cohort included all universal access Ontario Health Insurance Plan (OHIP) beneficiaries aged 18–80 from 1999 to 2018. The follow-up period was January 1, 1999 to December 31, 2018. This follow-up period was chosen based on availability of HCV testing data. Time zero for each individual was defined as the date they became eligible for follow-up if this occurred between 1999 and 2018, or January 1999 if they met the eligibility criteria prior to 1999. The maximum follow-up date was December 31, 2018, an individual’s death date, the date they turned 81, or the date they became ineligible for OHIP.

HCV Ab and HCV RNA laboratory test results from 1999 to 2018 were obtained from Public Health Ontario (PHO). PHO has 11 laboratories across Ontario, which provide services (e.g., HCV testing) to clinicians in primary care, hospitals, and public health units. HCV Ab testing was carried out in PHO laboratories using chemiluminescent microparticle immunoassay and HCV RNA testing used the Roche cobas® 6800 system [19,20]. The PHO HCV Ab and HCV RNA test result data were linked at ICES. ICES is an independent, non-profit research institute whose legal status under Ontario’s health information privacy law allows it to collect and analyze health care and demographic data, without consent, for health system evaluation and improvement. All data were accessed for research purposes on October 19, 2023, at which point data from all participants had already been anonymized with a unique individual identifier code. HCV tests with the same individual identifier code and test date were considered duplicates. When duplicate records differed by test type, RNA tests were prioritized over antibody tests because RNA testing can provide stronger evidence of current/active HCV infection. When multiple tests of the same type were recorded on the same day, positive test results were retained, as these were assumed to represent corrections of errors during laboratory analysis. Data for HCV risk factors were obtained, including HIV status, substance use disorder (SUD, injection drug use was not recorded), and birth cohort (to capture generations with an elevated HCV risk) [21]. HIV positivity was defined using the ICES HIV Database, which identifies HIV infection using a validated algorithm [22]. SUD was defined by a diagnostic code for drug dependence from the Discharge Abstract Database, the National Ambulatory Care Reporting System database, or the OHIP billing claims database. The diagnostic codes used to identify HIV and SUD are presented in S1 Table. The Registered Persons Database was used to define eligibility and birth cohort. Estimates of the annual population of Ontario aged 18–80 were found using the ICES POP database, which houses provincial and national population size estimates carried out by Statistics Canada. These datasets were linked using unique encoded identifiers and analyzed at ICES.

The annual count of HCV Ab, Ab+, RNA, and RNA+ tests in the PHO dataset were plotted to assess the impact of these HCV testing trends on each estimation method. To limit uninformative HCV testing data, only the earliest of consecutive HCV RNA+ tests from one individual were kept, and HCV Ab+ tests were removed if an individual had previously tested HCV Ab+.

### HCV incidence estimation methods

Four methods for estimating annual HCV incidence were considered. The methods differ in how they define the at-risk cohort and how they define incident HCV infection. The four methods will be referred to as the population-based method, the test-negative method, the RNA-based method, and the antibody-inclusive method. Table 1 provides an overview of each estimation method, including the at-risk cohort (denominator) HCV case definition (numerator), and a description of the incidence measurement.

**Table 1:** Description of four methods for estimating HCV incidence using extant testing data.

Method	At-risk denominator	Case definition (numerator)	Description of estimate
Population-based method	External estimate of the population of Ontario aged 18–80	Number of individuals meeting the HCV case definition (Ab+ < 2009; Ab+ or RNA+ 2010–2017; RNA+ 2018)	Cumulative incidence proportion
Test-negative method	Individuals that test HCV-	Individuals that test HCV+	Incidence among screened/high risk Ontarians(per 100,000 PY)
RNA-based method	Individuals that have not tested RNA+ , or whose most recent RNA test was negative	Individuals that test RNA+	Conservative estimate of HCV incidence rate in Ontario (per 100,000 PY)
Antibody-inclusive method	Individuals who have not tested Ab+ or RNA+ , or whose most recent RNA test was negative	Individuals who test Ab+ for the first time, or who test RNA+	Liberal estimate of HCV incidence rate in Ontario (per 100,000 PY)

**The population-based method** estimated the annual cumulative incidence of HCV by dividing the number of reported HCV cases in Ontario each year by the population of Ontario aged 18–80 during that year. The HCV case definitions used in this method adhered to the HCV case definitions used by PHO each year; the case definition used by PHO was an HCV Ab+ test result prior to 2009, an HCV Ab+ or RNA+ test result from 2009 to 2017, and an HCV RNA+ test result for 2018 [7,23]. The case count for each year was the number of individuals meeting the HCV case criteria in that year for the first time (e.g., each individual contributes a maximum of 1 case across all years in this method). The denominator of Ontarians aged 18–80 each year from 1999 to 2018 was identified using population estimates from Statistics Canada (the ICES POP database).

**The test-negative method** defined follow-up start as the date an individual tested HCV Ab- or HCV RNA-. Incident HCV infection was defined as: 1) testing HCV Ab+ or HCV RNA+ for those with a baseline HCV Ab- test result or 2) testing HCV RNA+ for those with a baseline HCV RNA- test result. The date an individual tested HCV+ was used as their date of infection. HCV+ individuals could re-enter the at-risk population if they subsequently tested HCV RNA-, indicating infection was cured or cleared. Follow-up for HCV-recovered individuals began on the date they tested HCV RNA-. In the case of individuals who tested HCV+ then HCV RNA- in the same year, their follow-up began on January 1 of the following year.

Poisson regression with no covariates was used to model HCV diagnosis events for each individual as a function of follow-up time since their most recent negative test date, providing estimates of annual HCV incidence-density (in units of cases per 100,000 person-years).

**The RNA-based method** used only HCV RNA test results and followed individuals that either: 1) never tested HCV RNA+ or 2) had cleared HCV infection (defined as an RNA+ test result followed by an RNA- test result). Eligibility for the RNA-based method began when individuals were aged 18–80 during 1999–2018, and incident HCV infection was defined as an RNA+ HCV test result. As described above, annual HCV incidence-density was calculated using Poisson regression, and individuals could re-enter the risk set in a later year if they tested HCV RNA-.

**The antibody-inclusive method** used the same cohort as the RNA-based method and defined incident HCV infection as the first recorded HCV Ab+ test for an individual, or an RNA+ test. A first-time antibody test was defined as the first recorded HCV Ab+ test result in the PHO dataset for an individual, provided that it was not preceded by an HCV RNA+ test result. First-time HCV Ab+ tests were used as cases regardless of whether they were confirmed by RNA testing. This was necessary because defining HCV Ab+ test results as cases conditional on the absence of subsequent RNA testing could introduce immortal time bias. As described above, annual HCV incidence-density was calculated using Poisson regression, and individuals could re-enter the risk set in a later year if they tested HCV RNA-.

### Testing method assumptions and validity

An analysis was carried out to investigate the impact of the population-based method adhering to the changing HCV case definition used by PHO (HCV Ab+ < 2009; HCV Ab+ or RNA+ 2009–2017; HCV RNA+ 2018) [7,23]. The effect of these changes in case definition on the population-based method was determined by estimating annual HCV incidence using HCV Ab+ as the case definition every year, then using HCV RNA+ as the case definition every year.

The generalizability of the test-negative method was evaluated by estimating the association (odds ratios) between established HCV risk factors (HIV, SUD, 1945–1975 birth cohort) and inclusion in the test-negative method cohort, using logistic regression. Additionally, the impact of reassigning the date of HCV infection to be the midpoint between the negative and positive test date was assessed, as this method has been used in previous test-negative cohorts to estimate HCV infection date more accurately [11,24].

Potential misclassification in the RNA-based and antibody-inclusive methods was quantified by determining the proportion of first-time Ab+ tests that were unconfirmed by RNA testing, the proportion followed by an RNA+ test, and the proportion followed by an RNA- test.

HCV testing data before January 1, 1999 were unavailable for the present analysis. This may have affected HCV incidence estimates because follow-up eligibility for each individual depended on HCV test results in prior years. To evaluate the robustness of incidence estimates for each method to the unavailability of HCV testing data before 1999, we conducted a sensitivity analysis that estimated annual HCV incidence for each method after excluding HCV testing data from before 2004.

This study received ethical approval from the Queen’s University Health Sciences & Affiliated Teaching Hospitals Research Ethics Board (reference number: 6039130).

## Results

**Fig 1:**
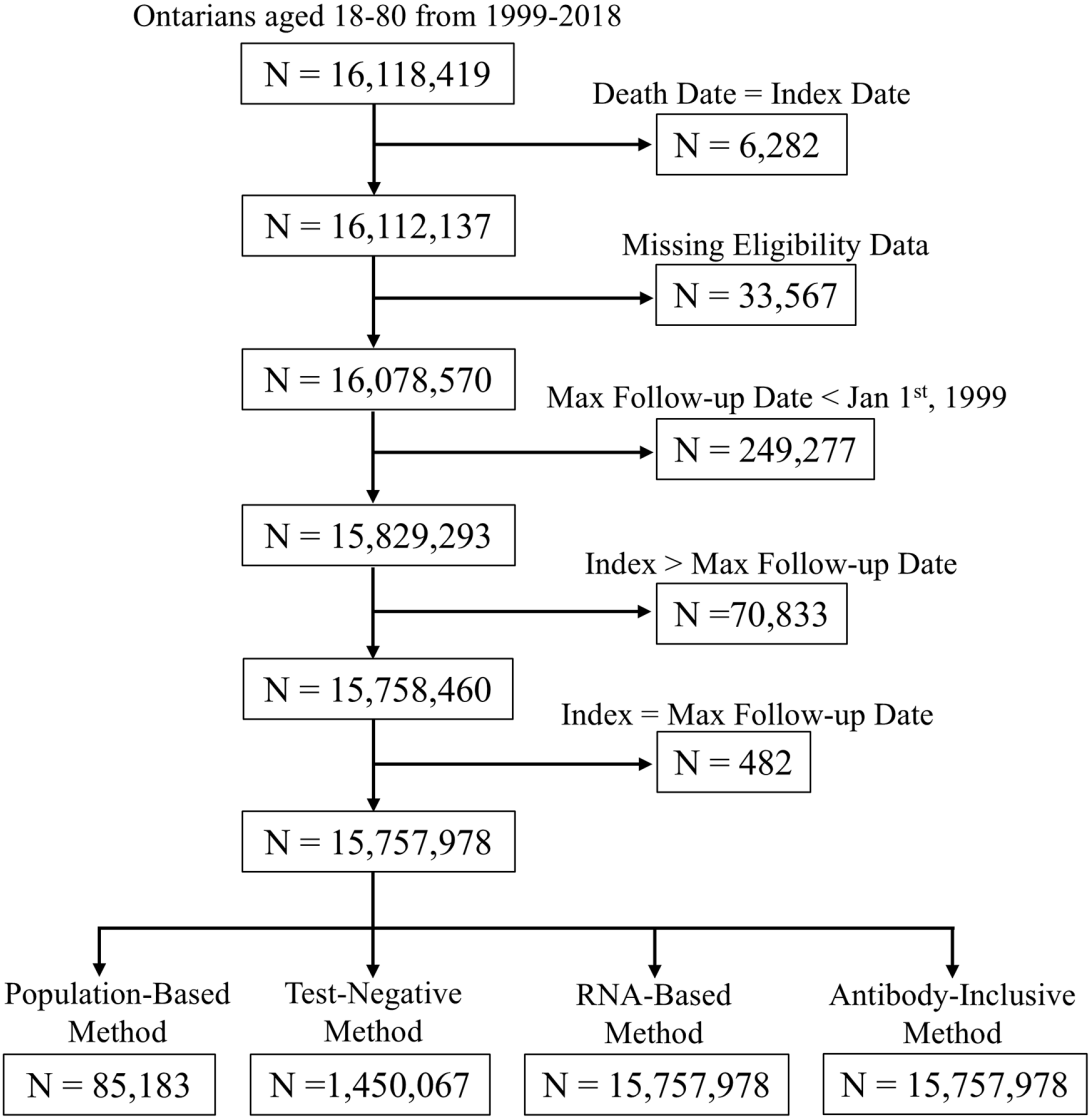
Flowchart of cohort creation for each HCV incidence estimation method. This figure shows the number of individuals excluded at each step of cohort derivation and the corresponding reasons for exclusion. The final cohort size for each method represents the population at risk used to measure HCV incidence. Since external estimates of population size were used in the population-based method, the cohort size shown reflects the number of individuals identified as HCV cases rather than a defined at-risk cohort.

**Fig 2:**
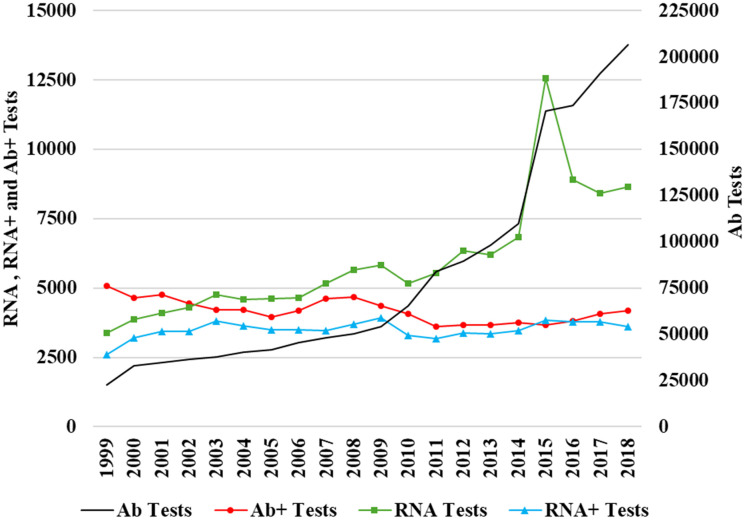
Annual frequency of HCV testing from 1999 to 2018 in Ontario, Canada. This figure plots the annual number of HCV antibody (Ab) tests (black, solid), Ab+ tests (red, circle), RNA tests (green, square), and RNA+ tests (blue, triangle). Consecutive HCV Ab+ tests and consecutive HCV RNA+ tests from the same individual are not included in this figure because these tests were not used in the analysis.

There were 16,118,419 individuals aged 18–80 from 1999–2018. After applying exclusion criteria, the study cohort consisted of 15,757,978 eligible individuals (Fig 1). Annual trends of HCV test results from the study cohort are reported in Fig 2 (annual counts of Ab tests, Ab+ tests, RNA tests, and RNA+ tests). Among the study cohort, there were 1,633,076 HCV Ab tests (83,605 Ab+) and 119,528 RNA tests (69,866 RNA+) used to estimate HCV incidence. While the frequency of Ab+ and RNA+ testing fluctuated moderately over follow-up, overall Ab and RNA testing increased until a spike in 2015, after which the rate of RNA testing dropped, and the rate of Ab testing continued to rise. S1 Fig reports these HCV testing trends without removing redundant tests that were not used in analysis (consecutive RNA+ and consecutive Ab+ test results from the same individual).

### HCV incidence estimates

**Fig 3:**
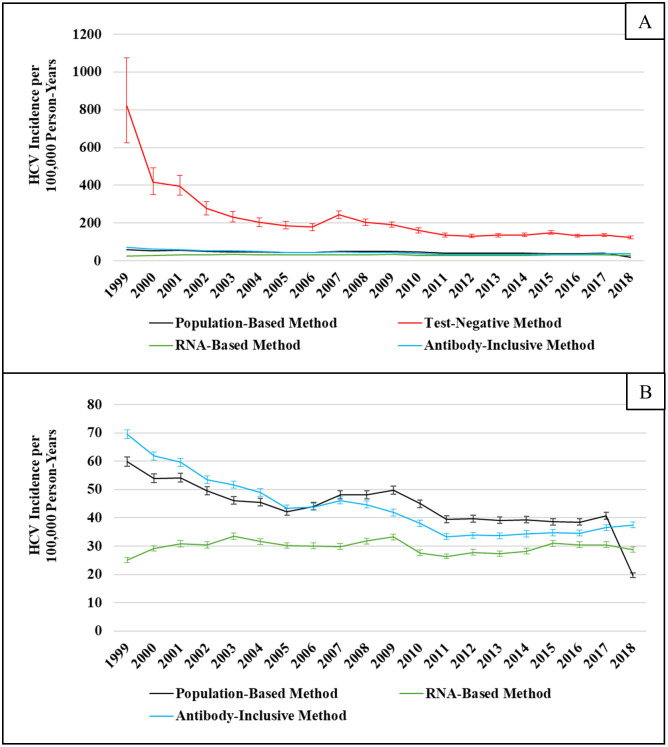
Annual HCV incidence estimates for each method. Panel A plots annual incidence estimates (and 95% confidence intervals) for the population-based method (black), the test-negative method (red), the RNA-based method (green), and the antibody-inclusive method (blue). Panel B shows the same data without plotting test-negative method estimates. Since the population-based method estimated cumulative incidence, incidence estimates for this method are in units of cases per 100,000 individuals.

HCV incidence estimates from 1999 to 2018 for each method are shown in Fig 3. Population-based method estimates of HCV incidence per 100,000 individuals declined from 59.8 in 1999 to 39.5 in 2011 (with an increase around 2009), remaining stable until a sharp decrease in 2018 to 19.8. Test-negative method estimates were higher than the other methods, and declined from 819.7 cases per 100,000 person-years (PY) in 1999 (with a spike in 2007), then plateaued at ~135 cases per 100,000 PY after 2011. RNA-based method estimates fluctuated around 30 cases per 100,000 PY, and antibody-inclusive method estimates declined from 69.5 cases per 100,000 PY in 1999 to 33.4 cases per 100,000 PY in 2011, then slowly rose until reaching 37.5 cases per 100,000 PY in 2018. The mean follow-up time was 5.6 ± 4.9 years for the test-negative method and 14.9 ± 6.3 years for both the RNA-based method and the antibody-inclusive method (follow-up data wasn’t used in the population-based method).

### Method assumptions and validity

**Fig 4:**
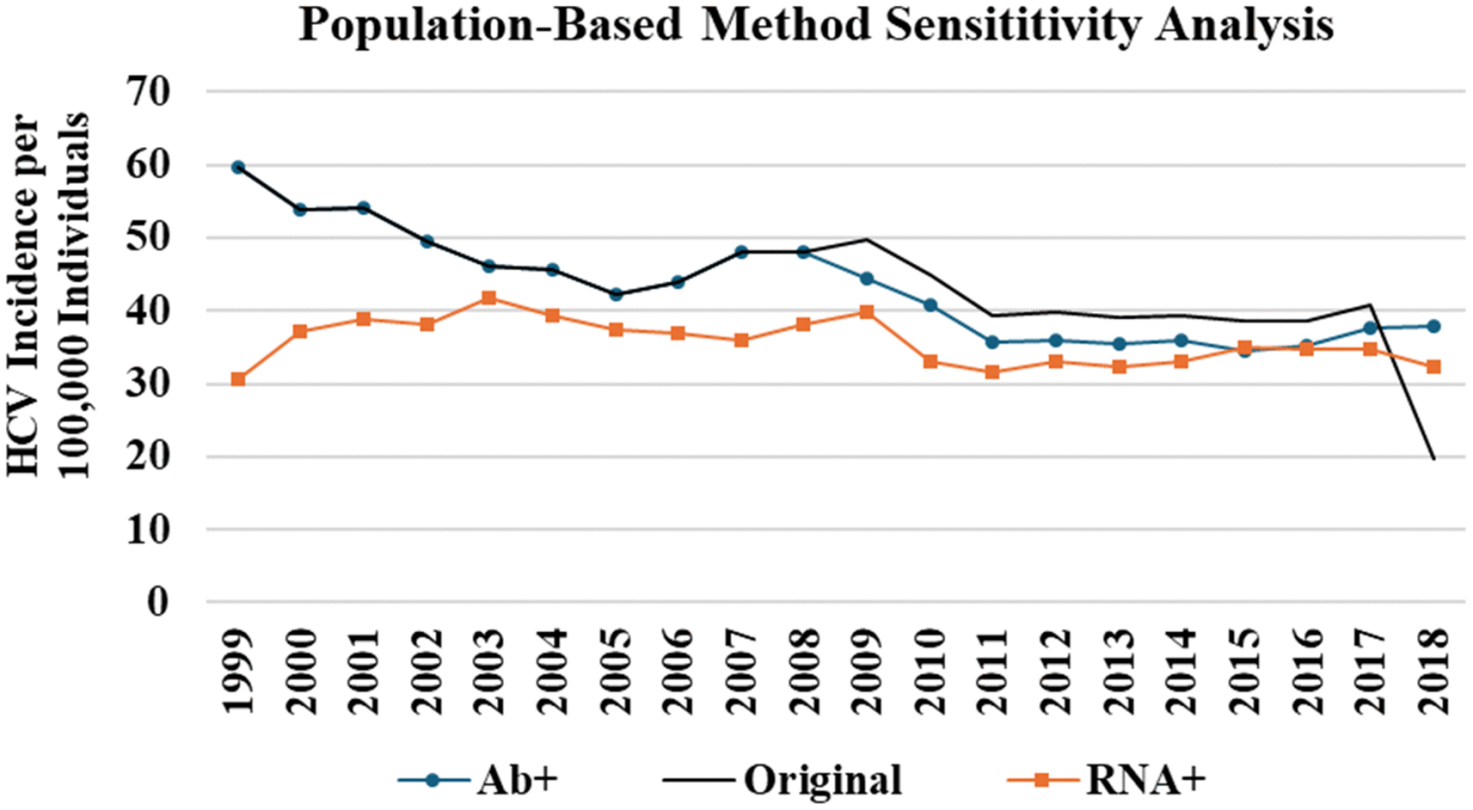
Effect of HCV case definition on population-based method estimates. This figure plots HCV incidence estimates for the population-based method when HCV Ab+ tests were used as the HCV case definition every year (blue, circle) and when RNA+ tests were used as the HCV case definition every year (orange, square). Estimates from the original analysis (black, solid) are also plotted, in which the population-based method adhered to HCV case definitions used by PHO each year (Ab+ < 2009; Ab+ or RNA+ 2009-2017; RNA+ in 2018).

Fig 4 reports population-based method estimates when using Ab+ testing to define HCV cases every year, and when using RNA+ testing to define HCV cases every year. The incidence estimates from the Ab+ analysis were lower than the original analysis from 2009 onward, except for 2018. Estimates from the RNA+ analysis were lower than the original in all years (except 2018), and became similar to the Ab+ analysis in 2015 and 2016.

**Fig 5:**
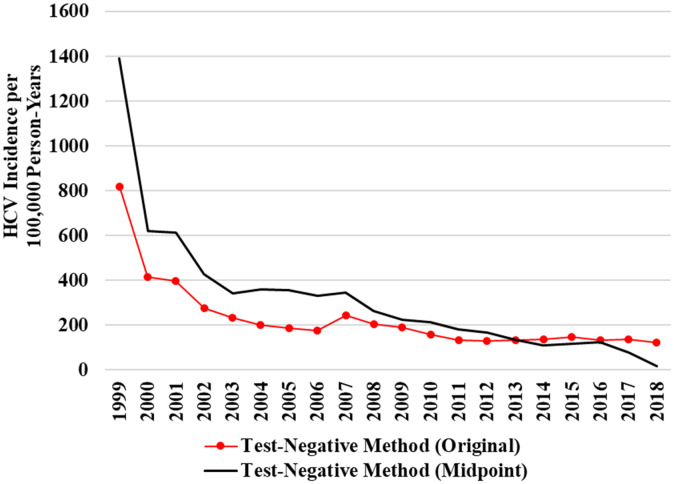
Effect of using the midpoint approach for the test-negative method. This figure plots HCV incidence estimates for the test-negative method when the date of HCV infection was reassigned to be the midpoint between the date an individual tested HCV+ and the date of the individual’s most recent HCV- test (black, solid), and estimates from the original analysis in which the test-negative method used the date an individual tested HCV+ to define infection date (red, circle).

The odds of being included in the test-negative method cohort were 10.5-fold higher for those positive for HIV (Odds Ratio (OR) = 10.5; 95% Confidence Interval (CI): 10.2, 10.7), 2.8-fold higher for those positive for SUD (OR = 2.77; 95% CI: 2.76, 2.78), and 4% lower for those born in the 1945–1975 birth cohort (OR = 0.964; 95% CI: 0.960, 0.967). Reassigning HCV infection date to be the midpoint between the HCV- and HCV+ test date inflated HCV incidence estimates early in follow-up, and diminished estimates later in follow-up (Fig 5).

There were 83,605 first-time HCV Ab+ test results in the present study. Of these, 50,921 (61%) were followed by a positive HCV RNA test result, 14,098 (17%) were followed by a negative HCV RNA test result, and 18,586 (22%) were not followed by an RNA test (e.g., unconfirmed HCV Ab+ tests).

**Fig 6:**
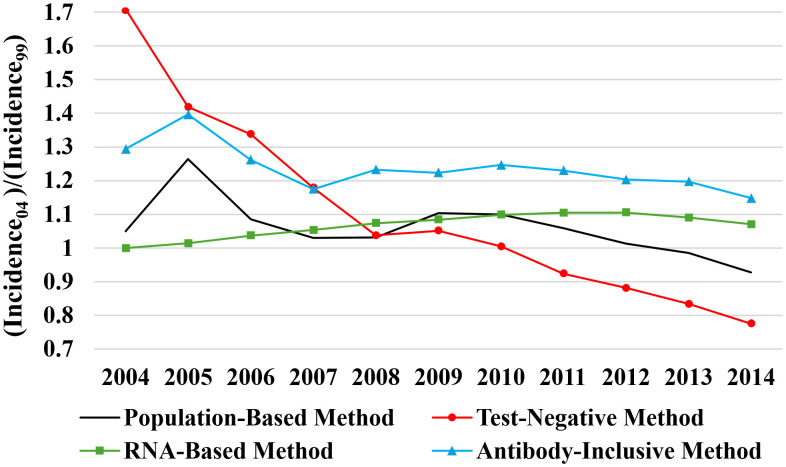
Effect of changing the start of follow-up from 1999 to 2004 on HCV incidence estimates for each method. This figure shows the ratio of incidence estimates with follow-up beginning in 2004 (Incidence₀₄) compared to 1999 (Incidence₉₉), quantifying how the absence of this data affected estimates from each method: population-based (black, solid), test-negative (red, circle), RNA-based (green, square), and antibody-inclusive (blue, triangle).

The effects of using 2004 as time zero rather than 1999 on HCV incidence estimates are reported in Fig 6. Changing time zero had a moderate effect on the population-based method, resulting in higher estimates early on and lower estimates later in follow-up; a much more severe effect of the same pattern was observed in the test-negative method estimates. Changing time zero to 2004 had only a minor impact on the HCV incidence estimates of the RNA-based method, while the antibody-inclusive method consistently had higher estimates after this change.

## Discussion

Our study is the first to use a population-based cohort to demonstrate four methods for estimating HCV incidence using extant HCV testing data. Primary concerns when using such data include non-completion of the HCV testing cascade and differentiating incident and prevalent HCV infections while also preserving generalizability.

The general increase in HCV testing over follow-up can partly be explained by the rise in the number of Ontarians aged 18–80 over follow-up, and likely also an increased number of individuals offered HCV screening as opioid use increased over this time period. [25] More granular trends can be explained by the release of updated HCV management guidelines in 2005 and 2009, while the large increase in testing in 2015 coincided with the inclusion of the 1945–1975 birth cohort in risk-based HCV screening, and the wide availability of Direct Acting Antivirals as an effective and well-tolerated treatment to cure HCV infection. [14,26–29]

**The population-based method** used simple calculations and only required aggregate annual HCV testing data, while the other methods required statistical software packages for regression analysis. Changes in the Ontario HCV case definition greatly impacted population-based method estimates, limiting generalizability to regions that changed their HCV case definition in different years, including the USA and the European Union. [30–32] Shifts in the HCV case definition over follow-up also made the population-based method less suitable for evaluating trends in HCV incidence than the other three methods, which used consistent HCV case definitions over follow-up. Population-based method incidence estimates likely dropped in 2018 because the case definition for incident HCV infection was changed to HCV RNA+ in 2018, and a large proportion of individuals that tested RNA+ in 2018 tested Ab+ in a previous year and were removed from the risk set. Therefore, the 2018 case count for the population-based method only included the subset of individuals that tested both Ab+ and RNA+ in 2018, or who tested RNA+ in 2018 with no prior Ab+ tests.

**The test-negative method** only included HCV cases that transitioned from HCV- to HCV+ while under observation, providing strong evidence that these cases were incident infections. The other three methods assumed individuals were HCV- if they had not tested HCV+ , potentially increasing the rate of HCV misclassification. The generalizability of test-negative method incidence estimates was likely limited due to a higher prevalence of HIV and SUD in the test-negative cohort compared to the cohorts used for the other three methods, since inclusion in the test-negative cohort was conditional on being offered risk-based HCV screening [13].

The cohort size for the test-negative method started very small because the cohort each year only consisted of individuals that tested HCV- in that year or a prior year (S2 Fig plots the annual cohort size and case count for each method). Low sample size early in follow-up led to large error in HCV incidence estimates, which could explain why HCV incidence estimates for the test-negative method were implausibly high early in follow-up, but began to stabilize over time. Conversely, sample size was high in all years for the other three methods, leading to consistently low error in HCV incidence estimates in all years of follow-up. This likely also explains why the test-negative method was more susceptible to changing time zero from 1999 to 2004 compared to the other methods.

The test-negative method had the advantage of being able to assign the date of HCV infection to be the midpoint between HCV- and HCV+ test dates. This method has been used in previous studies to improve precision in the classification of HCV infection date by assuming that the average date of infection occurred halfway between negative and positive HCV test dates [11,24]. While the true date of infection must fall in this window, choosing the midpoint to represent infection date risks misclassification of HCV infection date, especially when the duration between the negative and positive test is long (average of 4 years in the present study). This change inflated HCV incidence estimates earlier in follow-up and diminished them later in follow-up (Fig 5). This is because cases were only reassigned from later years to earlier years; 1999 gained HCV cases while losing none, and the ratio of lost cases to gained cases increased for each year of follow-up until 2018 when HCV cases were only lost. It is therefore important to recognize that when HCV testing data is only available for a specific window of time, reassigning infection dates to the midpoint between an HCV- and HCV+ test could lead to the potentially false conclusion that the incidence of HCV is decreasing.

**The RNA-based method** used a generalizable cohort, prioritized specificity of HCV classification, and was more resistant to changing time zero than the other three methods. However, HCV incidence was underestimated because 22% of HCV Ab+ tests were unconfirmed by an RNA test, and a proportion of these individuals would have tested RNA+. This proportion may have been large given that most untreated HCV infections become chronic, and HCV treatment cannot be prescribed without testing HCV RNA+ [10,13,16]. This is further supported by the fact that among HCV Ab+ tests in the present study that *were* followed by an RNA test, it was much more common for this RNA test to be positive (N = 50,921) than negative (N = 14,098).

**The antibody-inclusive method** prioritized sensitivity by including all first-time HCV Ab+ test results as cases, which likely led to overestimation of HCV incidence. The antibody-inclusive method assumes all unconfirmed Ab+ tests would have tested RNA+, while the RNA-based method is conservative in assuming none of them would have tested HCV RNA+. In settings with a low rate of unconfirmed HCV Ab+ tests, the RNA-based method would likely provide more accurate estimates of HCV incidence. The test-negative method is less impacted by unconfirmed HCV Ab+ tests because this method required Ab+ tests to be preceded by an Ab- test, which provides evidence of incident HCV infection without a confirmatory RNA test. Trends in HCV incidence estimates from the antibody-inclusive method resembled estimates published by PHO between 1999 and 2018 (S3 Fig), although these PHO estimates use extant HCV data and are therefore also affected by the biases considered in this study [9,33–35].

Private laboratories are authorized to carry out HCV Ab screening, but are not required to submit HCV Ab- test results to PHO [19]. The absence of HCV Ab- test results from private laboratories in the present study could have reduced the denominator of the test-negative method cohort. Additionally, the absence of these test results could impact generalizability of results to individuals receiving HCV testing in private laboratories.

Reducing the global burden of HCV involves preventing new HCV infections (incident infections) and curing existing HCV infections (prevalent infections) [36]. HCV incidence aims to measure the rate of new HCV infections in a population over time, and is therefore a useful metric to measure the success of preventative interventions. Prospective cohort studies are the gold standard for measuring HCV incidence because this method differentiates incident and prevalent HCV infections with high accuracy [3,5]. The use of extant HCV testing data is accepted by the WHO as a pragmatic alternative for measuring HCV incidence when prospective cohort studies are infeasible, with acknowledgement of important limitations [2,3]. Extant testing data provides the date of HCV diagnosis, which can be an imperfect proxy for the date of HCV infection if chronic HCV infection persists for years before diagnosis [10]. While the date of HCV infection can be approximated using the duration between an HCV- test date and HCV+ test date (e.g., the midpoint), this method is imprecise when the mean duration between negative and positive tests is long (4 years in the present study).

The WHO strategy to reduce the global burden of HCV also emphasizes the need for programs that increase the rate of HCV screening and the availability and uptake of direct-acting antiviral therapy [36]. The success of such programs is ideally measured using repeated estimates of HCV viremic prevalence, which is the proportion of individuals in a population that are HCV RNA+ at a point in time [5]. Extant HCV testing data is frequently used to measure HCV prevalence, likely because HCV RNA+ test results can provide evidence of prevalent HCV infection [37,38]. However, this method will underestimate HCV prevalence in regions such as Canada, where it is estimated that 25% of chronic HCV infections are undiagnosed and therefore absent in repositories of extant testing data [39]. Multiple approaches have been used in past studies to incorporate undiagnosed chronic HCV cases into estimates of HCV prevalence, such as back-calculation modelling and machine learning to impute undiagnosed HCV cases based on administrative health data [39,40].

The utility of the methods discussed in the present study will likely vary across contexts depending on HCV burden, mode of infection, and HCV testing and reporting practices. These methods are not applicable in regions that do not have HCV surveillance systems that comprehensively record positive HCV test results, such as some low-middle income countries [41]. The generalizability of the test-negative method could potentially be higher in regions where universal HCV screening has been adopted, because individuals that test HCV- would likely better represent the underlying population rather than high-risk sub-groups. Universal HCV screening has been implemented in populations where medical procedures are a major mode of HCV transmission, unlike Canada where HCV primarily spreads through high risk behaviors such as IDU. [42] The test-negative method relies on cataloging of negative HCV test results: a report on HCV elimination efforts in high burden countries found that registries of HCV test results were only kept in 55% of these countries [41]. In regions like the USA with HCV surveillance systems and where testing practices are shifting from risk-based to universal HCV screening, the test-negative method could become a valid approach for measuring HCV incidence [43].

The performance of the RNA-based and antibody-inclusive methods will likely vary depending on the rate of unconfirmed HCV Ab+ test results, which is especially high in settings where HCV RNA testing is not commonly available [44]. The RNA-based method would likely perform poorly in these regions, making the antibody-inclusive method preferable. However, as point-of-care RNA testing is adopted in more regions, the applicability of the RNA-based method could improve [41,45]. Additionally, HCV laboratory testing protocols in Ontario are moving towards reflexive HCV RNA testing on blood samples that are HCV Ab+, which could substantially decrease the rate of unconfirmed HCV Ab+ tests and improve the applicability of the RNA-based method [19].

## Conclusion

The present study compared four methods for estimating the incidence of HCV using extant HCV testing data. The population-based method was efficient but more prone to misclassification than the test-negative method. The test-negative method, however, provided incidence estimates that may only be generalizable to high-risk populations. HCV incidence estimates were likely more generalizable for the RNA-based and antibody-inclusive methods. Unconfirmed HCV Ab+ tests led HCV incidence to be underestimated in the RNA-based method and overestimated in the antibody-inclusive method. These results provide much needed guidance for measuring progress towards WHO HCV incidence targets in regions that rely on extant HCV testing data.

## Supporting information

S1 Fig Annual frequency of HCV testing with inclusion of uninformative test results. This figure plots the number of HCV Ab tests (black, solid), Ab+ tests (red, circle), RNA tests (green, square), and RNA+ tests (blue, triangle) each year, including consecutive HCV Ab+ results and consecutive RNA+ results from the same individual.
(TIF)

S2 Fig Annual cohort size and case count each method. This figure plots the annual cohort size (blue, solid) and case count (orange, square) used for the population-based method (A), test-negative method (B), RNA-based method (C), and antibody-inclusive method (D).
(TIF)

S3 Fig HCV incidence estimates from the antibody-inclusive method compared to published estimates. This figure plots annual HCV incidence estimates for the antibody-inclusive method (blue, triangle) and HCV incidence estimates for the province of Ontario published by Public Health Ontario (purple, solid), from 2002 to 2018.
(TIF)

S1 Table Diagnostic codes used to identify diagnoses of HIV and SUD using ICES data. (DOCX)

S2 Table HCV incidence estimates and confidence intervals for the test-negative method, the RNA-based method, and the antibody-inclusive method. (DOCX)
