# Supplementary figures and images for: Four methods for estimating hepatitis C incidence using extant testing data
Source: PLoS One. 2026 Jun 10;21(6):e0335115. doi: 10.1371/journal.pone.0335115 (PMC13252766; doi:10.1371/journal.pone.0335115)

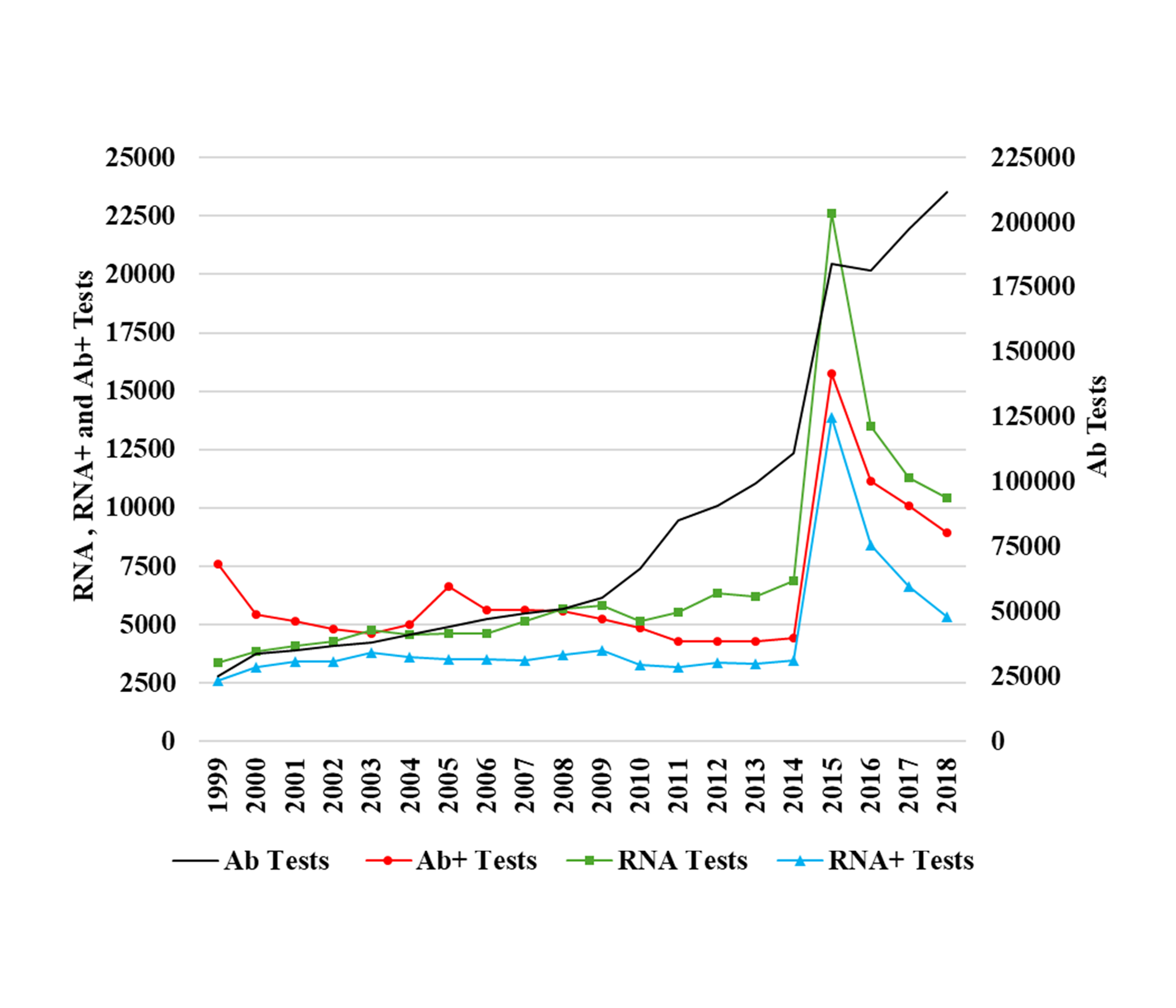

Supplement: S1 Fig — This figure plots the number of HCV Ab tests (black, solid), Ab+ tests (red, circle), RNA tests (green, square), and RNA+ tests (blue, triangle) each year, including consecutive HCV Ab+ results and consecutive RNA+ results from the same individual. (TIF) [file pone.0335115.s001.tif]

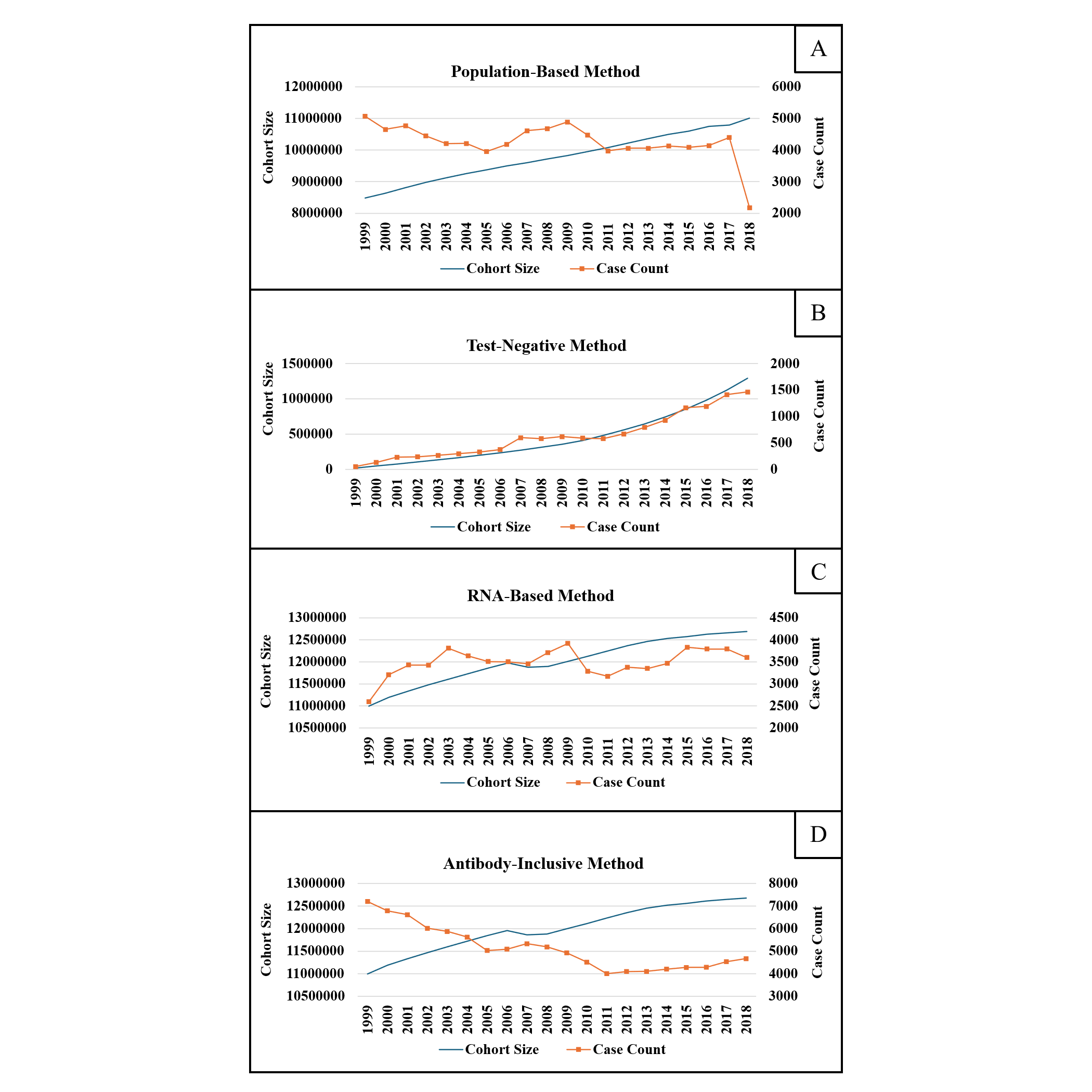

Supplement: S2 Fig — This figure plots the annual cohort size (blue, solid) and case count (orange, square) used for the population-based method (A), test-negative method (B), RNA-based method (C), and antibody-inclusive method (D). (TIF) [file pone.0335115.s002.tif]

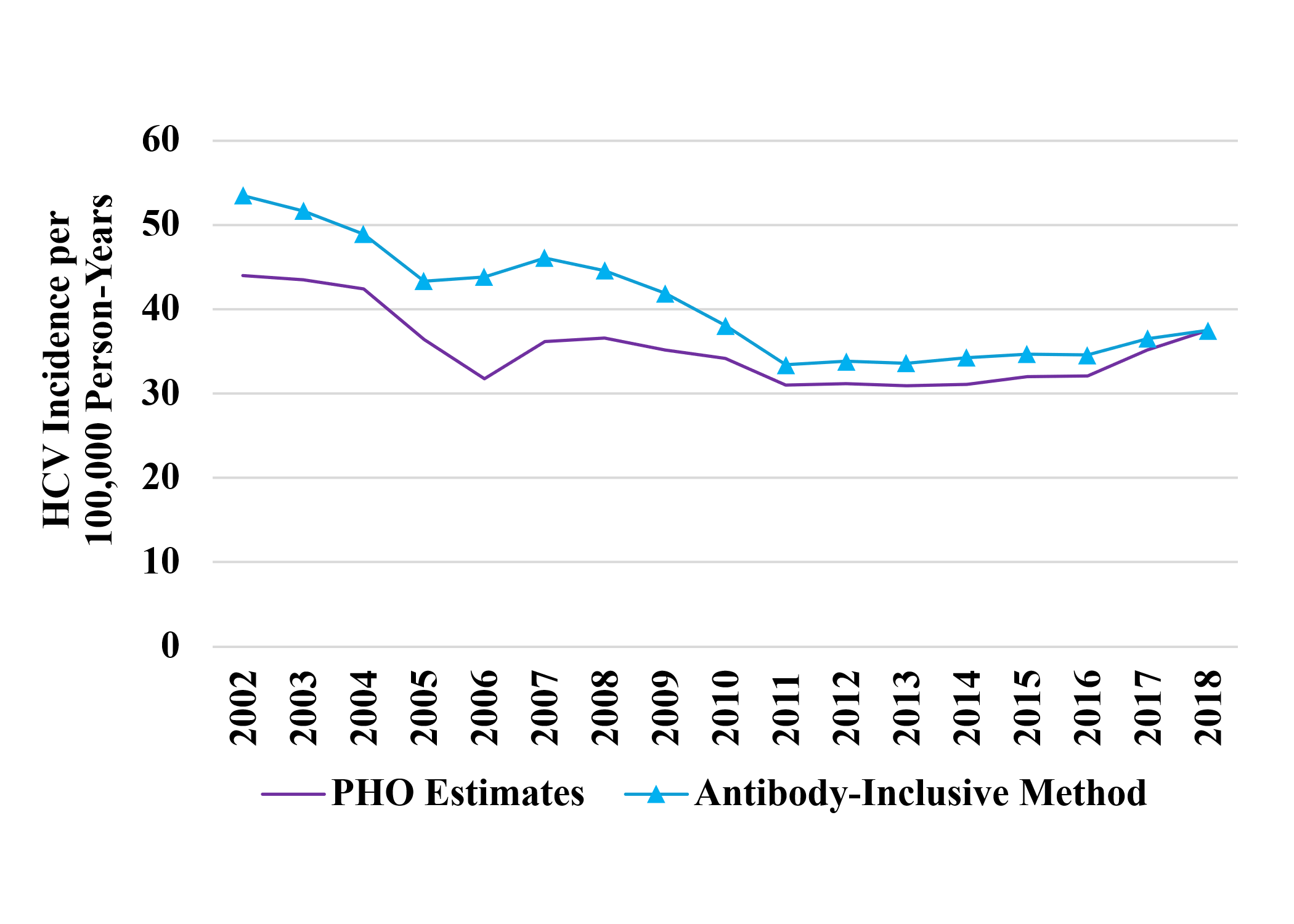

Supplement: S3 Fig — This figure plots annual HCV incidence estimates for the antibody-inclusive method (blue, triangle) and HCV incidence estimates for the province of Ontario published by Public Health Ontario (purple, solid), from 2002 to 2018. (TIF) [file pone.0335115.s003.tif]
