# Supplementary material for: Four methods for estimating hepatitis C incidence using extant testing data
Source: PLoS One. 2026 Jun 10;21(6):e0335115. doi: 10.1371/journal.pone.0335115 (PMC13252766; doi:10.1371/journal.pone.0335115)
Supplement: S1 Table — (DOCX) [file pone.0335115.s004.docx]

| **S1 Table.** Diagnostic codes used to identify diagnoses of HIV and SUD using ICES data | |
| --- | --- |
| Variable | Definition |
| HIV | 3 physician claims in 3 years  ICD-9 codes 042–044  ICD-10 codes B20–B24 |
| SUD | SUD was defined by any of the following diagnosis/service codes.  ICD-9 Codes: 2920, 2921.x, 2922, 2928.x, 2929, 3040.x, 3041.x, 3042.x, 3044.x, 3045.x, 3046.x, 3047.x, 3048.x, 3049.x, 3053.x , 3054.x, 3055.x, 3056.x, 3057.x, 3058.x, 3059.x, 6483.x, 9650.x, 9670, 9671, 9674, 9675, 9676, 9678, 9679, 9683, 9691, 9692, 9694, 9695, 9696, 9697, 9698, 9699, 9708, 9709  ICD-10: F110, F111, F112, F113, F114, F115, F116, F117, F118, F119, F130, F131, F132, F133, F134, F135, F136, F137, F138, F139, F140, F141, F142, F143, F144, F145, F146, F147, F148, F149, F150, F151, F152, F153, F154, F155, F156, F157, F158, F159, F160, F161, F162, F163, F164, F165, F166, F167, F168, F169, F190, F191, F192, F193, F194, F195, F196, F197, F198, F199, O35501, O35503, O35509, R781, R782, R783, R784, T401, T402, T4020, T4021, T4022, T4023, T4028, T403, T404, T4040, T4041, T4048, T405, T406, T408, T409, T411, T423, T424, T426, T427, T436, X41, X42, X61, X62, Y11, Y12, Z503, Z715, Z722, Z8641  OHIP: 304, 292  DSM-4: Provisional code 4  DSM-5: Provisional code 16 |
