# Supplementary material for: Four methods for estimating hepatitis C incidence using extant testing data
Source: PLoS One. 2026 Jun 10;21(6):e0335115. doi: 10.1371/journal.pone.0335115 (PMC13252766; doi:10.1371/journal.pone.0335115)
Supplement: S2 Table — (DOCX) [file pone.0335115.s005.docx]

| **S2 Table.** HCV incidence estimates and confidence intervals for the test-negative method, the RNA-based method, and the antibody-inclusive method | | |
| --- | --- | --- |
| Test-Negative Method | | |
| Year | Point Estimate | 95% CI |
| 1999 | 819.7 | 624.7, 1075.8 |
| 2000 | 415.4 | 349.8, 493.3 |
| 2001 | 396.4 | 348.3, 451.2 |
| 2002 | 276.2 | 243.2, 313.8 |
| 2003 | 232.4 | 206.0, 262.1 |
| 2004 | 202.2 | 180.3, 226.7 |
| 2005 | 186.4 | 167.4, 207.7 |
| 2006 | 177.5 | 160.4, 196.5 |
| 2007 | 243.0 | 224.3, 263.3 |
| 2008 | 204.1 | 188.2, 221.4 |
| 2009 | 191.0 | 176.5, 206.6 |
| 2010 | 159.7 | 147.3, 173.1 |
| 2011 | 134.3 | 123.8, 145.7 |
| 2012 | 130.8 | 121.2, 141.1 |
| 2013 | 134.7 | 125.7, 144.4 |
| 2014 | 136.2 | 127.7, 145.3 |
| 2015 | 149.1 | 140.8, 157.9 |
| 2016 | 131.8 | 124.6, 139.6 |
| 2017 | 136.6 | 129.6, 143.9 |
| 2018 | 123.3 | 117.1, 129.8 |
| RNA-Based Method | | |
| Year | Point Estimate | 95% CI |
| 1999 | 25.0 | 24.1, 26.0 |
| 2000 | 29.2 | 28.2, 30.2 |
| 2001 | 30.8 | 29.8, 31.9 |
| 2002 | 30.4 | 29.4, 31.5 |
| 2003 | 33.5 | 32.4, 34.6 |
| 2004 | 31.6 | 30.6, 32.6 |
| 2005 | 30.2 | 29.2, 31.2 |
| 2006 | 30.1 | 29.1, 31.1 |
| 2007 | 29.9 | 28.9, 30.9 |
| 2008 | 31.8 | 30.8, 32.8 |
| 2009 | 33.2 | 32.2, 34.3 |
| 2010 | 27.6 | 26.7, 28.6 |
| 2011 | 26.4 | 25.5, 27.3 |
| 2012 | 27.8 | 26.9, 28.8 |
| 2013 | 27.4 | 26.5, 28.3 |
| 2014 | 28.2 | 27.3, 29.2 |
| 2015 | 31.0 | 30.0, 32.0 |
| 2016 | 30.5 | 29.6, 31.5 |
| 2017 | 30.5 | 29.5, 31.5 |
| 2018 | 28.8 | 27.9, 29.8 |
| Antibody-inclusive Method | | |
| Year | Point Estimate | 95% CI |
| 1999 | 69.5 | 67.9, 71.1 |
| 2000 | 61.8 | 60.4, 63.3 |
| 2001 | 59.5 | 58.1, 61.0 |
| 2002 | 53.5 | 52.2, 54.9 |
| 2003 | 51.7 | 50.4, 53.0 |
| 2004 | 48.9 | 47.7, 50.2 |
| 2005 | 43.4 | 42.2, 44.6 |
| 2006 | 43.9 | 42.7, 45.1 |
| 2007 | 46.1 | 44.9, 47.4 |
| 2008 | 44.6 | 43.4, 45.9 |
| 2009 | 41.9 | 40.7, 43.1 |
| 2010 | 38.0 | 37.0, 39.2 |
| 2011 | 33.4 | 32.4, 34.4 |
| 2012 | 33.8 | 32.8, 34.9 |
| 2013 | 33.6 | 32.6, 34.7 |
| 2014 | 34.3 | 33.3, 35.4 |
| 2015 | 34.7 | 33.7, 35.8 |
| 2016 | 34.6 | 33.6, 35.6 |
| 2017 | 36.5 | 35.5, 37.6 |
| 2018 | 37.5 | 36.4, 38.6 |
