# Supplementary material for: Four methods for estimating hepatitis C incidence using extant testing data
Source: PLoS One. 2026 Jun 10;21(6):e0335115. doi: 10.1371/journal.pone.0335115 (PMC13252766; doi:10.1371/journal.pone.0335115)
Supplement: Attachment — Submitted filename: Reviewer responses.docx [file pone.0335115.s007.docx]

**Reviewer Responses -** **Four methods for estimating hepatitis C incidence using extant testing data**

Reviewer #1: Four methods for estimating hepatitis C incidence using extant testing data

Dear Authors,
Below are detailed comments organized by manuscript section to guide your revisions and enhance clarity, transparency, and reproducibility.

The abstract is well structured and summarizes the study clearly.
However, several improvements would strengthen its clarity and scientific impact:
1. Background: Add one introductory sentence linking HCV incidence estimation to its global public health relevance.

**Thank you for this comment, relevance of this work is now framed in the context of HCV elimination goals set by the World Health Organization. This framework was chosen to align with previous publications investigating measurement of HCV incidence.^1-3^**

**“Accurate estimation of HCV incidence is crucial for measuring progress towards HCV elimination targets set by the World Health Organization (WHO).”**

1. **Mitchell HD, Clancy I, De Angelis D, et al. Using modelling and public health surveillance data to evidence progress towards the WHO incidence target for hepatitis C elimination in people who inject drugs - a review of complementary approaches in the United Kingdom. Int J Drug Policy. Dec 2025;146:105059. doi:10.1016/j.drugpo.2025.105059**
2. **Artenie A, Luhmann N, Lim AG, et al. Methods and indicators to validate country reductions in incidence of hepatitis C virus infection to elimination levels set by WHO. Lancet Gastroenterol Hepatol. Apr 2022;7(4):353-366. doi:10.1016/S2468-1253(21)00311-3**
3. **Popovic N, Williams A, Perinet S, et al. National Hepatitis C estimates: Incidence, prevalence, undiagnosed proportion and treatment, Canada, 2019. Can Commun Dis Rep. Nov 3 2022;48(11-12):540-549. doi:10.14745/ccdr.v48i1112a07**

2. Methods: Clarify the data source (ICES, Ontario) and briefly mention the statistical method used (e.g., Poisson regression).

**ICES is an independent, non-profit research institute whose legal status under Ontario’s health information privacy law allows it to collect and analyze health care and demographic data, without consent, for health system evaluation and improvement.**

**This is now reflected more clearly in the abstract:**

**“Methods: Using databases linked at ICES ­– a repository of administrative health data for Ontario residents – we constructed a cohort of 15.8 million Ontarians aged 18–80 between 1999 and 2018 to estimate annual HCV incidence using four methods”**

**The methods section of the abstract now emphasizes that the population-based method used simple calculations to estimate HCV incidence, while the other three methods used Poisson regression.**

**“The population-based method calculated HCV incidence as the number of HCV cases confirmed through HCV testing each year divided by population size estimates, while Poisson regression was used in the other three incidence estimation methods”**

3. Results: Use a short opening phrase (e.g., Results showed distinct incidence patterns across methods) and simplify wording such as influenced by → sensitive to.

**Incidence estimates from each method in the results section of the abstract are now prefaced by the following statement:**

**“Distinct patterns of HCV incidence were found across the estimation methods…”**

**The following change was made:**

**“Population-based estimates were sensitive to changes in…”**

4. Conclusion: Strengthen the final statement by linking it to public health applications or surveillance implications.

**The concluding statement ties the significance of the results back into the evaluation of achievement of WHO HCV incidence targets”**

**“These new findings illustrate the influence of cohort definition and HCV case definition when estimating HCV incidence using extant testing data, which will support accurate measurement of progress towards WHO HCV elimination goals.”**

Peer Review Introduction Section
1. Add a recent statistic on global HCV prevalence to strengthen the topic’s importance.

**The introduction now opens with the following statistics:**

**“Approximately 242,000 individuals died from hepatitis C (HCV) infection globally in 2022 and around one million new HCV cases occur each year.^1^”**

1. **The World Health Organization. (2025, 25 July 2025). Hepatitis C. Retrieved January 22 from https://www.who.int/news-room/fact-sheets/detail/hepatitis-c**

2. Include a practical example of a health intervention that relies on incidence estimation.

**The authors agree that early discussion of the practical importance of HCV incidence estimation would strengthen the introduction. The following sentences were added to emphasize this importance and reference existing work in this area:**

**“Updated global HCV elimination targets were announced by the WHO in 2023, which included a reduction in HCV incidence to ≤5 new annual HCV cases per 100,000 adults by 2030.^1^ Since the quantity and quality of HCV testing data varies across regions, several approaches have been proposed for measuring progress towards this HCV incidence target.^1-4^”**

1. **The World Health Organization. Guidance for country validation of viral hepatitis elimination and path to elimination. Global HIV HaSPH; 2023.** [**https://www.who.int/publications/i/item/9789240078635**](https://www.who.int/publications/i/item/9789240078635)
2. **The World Health Organization. Interim guidance for country validation of viral hepatitis elimination. Global HIV HaSPH; 2021.** [**https://www.who.int/publications/i/item/9789240028395**](https://www.who.int/publications/i/item/9789240028395)
3. **Mitchell HD, Clancy I, De Angelis D, et al. Using modelling and public health surveillance data to evidence progress towards the WHO incidence target for hepatitis C elimination in people who inject drugs - a review of complementary approaches in the United Kingdom. Int J Drug Policy. Dec 2025;146:105059. doi:10.1016/j.drugpo.2025.105059**
4. **Artenie A, Luhmann N, Lim AG, et al. Methods and indicators to validate country reductions in incidence of hepatitis C virus infection to elimination levels set by WHO. Lancet Gastroenterol Hepatol. Apr 2022;7(4):353-366. doi:10.1016/S2468-1253(21)00311-3**

3. Briefly hint at the types of strengths and limitations of the methods.

**We appreciate the Reviewer’s comment, and would like to note that the strengths and limitations of each method are described in the introduction:**

**Paragraph 3 mentions that the population-based method is efficient but prone to misclassification, and that the test negative method is less prone to misclassification, possibly at the cost of generalizability.**

**Paragraph 4 mentions that the RNA-based method likely improves generalizability may underestimate HCV incidence due to unconfirmed Ab+ tests**

**Paragraph 5 mentions that the antibody-inclusive method makes use of unconfirmed Ab+ tests but may overestimate HCV incidence**

4. Add a concluding sentence explaining how the comparison can improve public health policies.

**The introduction now includes a concluding paragraph to reiterate that many regions rely on extant HCV testing data to measure progress towards HCV elimination goals, without necessarily understanding the limitations of this data source:**

**“When prospective cohort studies are infeasible, regions often rely on extant HCV testing data to measure progress towards WHO HCV elimination targets.^1-3^ HCV incidence can be measured using extant HCV testing data through a variety of approaches, the strengths and limitations of which are poorly understood. Therefore, the objective of this study was to use extant HCV testing data to estimate the annual incidence of HCV from 1999 to 2018 in Ontario using four different methods, and comment on the generalizability and potential biases for each method.”**

1. **The World Health Organization. Interim guidance for country validation of viral hepatitis elimination. Global HIV HaSPH; 2021.** [**https://www.who.int/publications/i/item/9789240028395**](https://www.who.int/publications/i/item/9789240028395)
2. **The World Health Organization. Guidance for country validation of viral hepatitis elimination and path to elimination. Global HIV HaSPH; 2023.** [**https://www.who.int/publications/i/item/9789240078635**](https://www.who.int/publications/i/item/9789240078635)
3. **Artenie A, Luhmann N, Lim AG, et al. Methods and indicators to validate country reductions in incidence of hepatitis C virus infection to elimination levels set by WHO. Lancet Gastroenterol Hepatol. Apr 2022;7(4):353-366. doi:10.1016/S2468-1253(21)00311-3**

Peer Review Comment Section: Data Source
Data sources are clearly presented and well-linked across databases. Unique identifiers prevent duplication, and coverage from all 11 PHO labs is a key strength. Minor clarifications are needed regarding duplication checks, data validation, and potential selection bias.

**The following statement was added to provide clarity on how duplicate tests were defined, and how specific duplicate test scenarios were handled at the data cleaning stage:**

**“HCV tests with the same individual identifier code and test date were considered duplicates. When duplicate records differed by test type, RNA tests were prioritized over antibody tests because RNA testing can provide definitive evidence of current/active infection. When multiple tests of the same type were recorded on the same day, positive test results were retained, as these were assumed to represent corrections of errors during laboratory analysis.”**

**Gaps in ascertainment of HCV testing data are now discussed in the following paragraph**

**Private laboratories are authorized to carry out HCV Ab screening, but are not required to submit HCV Ab- test results to PHO.^1^ The absence of HCV Ab- test results from private laboratories in the present study could have reduced the denominator of the test-negative method cohort. Additionally, the absence of these test results could impact generalizability of results to individuals receiving HCV testing in private laboratories.**

1. **Ontario Agency for Health Protection and Promotion (Public Health Ontario). Hepatitis C – Diagnostic Serology. Accessed January 10th, 2026. https://www.publichealthontario.ca/en/Laboratory-Services/Test-Information-Index/Hepatitis-C-Diagnostic-Serology**

Peer Review Comment Section: HCV Incidence Estimation Methods
Although the Results and Discussion describe the four methods well, the Methods section still needs a brief explanation of how incidence was calculated for each approach and how comparability was ensured. Adding these details would improve methodological clarity and reproducibility.

**The following paragraph was added to the description of the test-negative method to clarify how HCV incidence was estimated**

**“Poisson regression with no covariates was used to model HCV diagnosis events for each individual as a function of follow-up time since their most recent negative test date, providing estimates of annual HCV incidence-density (in units of cases per 100,000 person-years).”**

**A brief statement was added after the RNA-based and antibody-inclusive methods to indicate that incidence estimation using Poisson regression was consistently used in these three methods:**

**“As described above, annual HCV incidence density was calculated using Poisson regression, and individuals could re-enter the risk set in a later year if they tested HCV RNA-.”**

**The manual calculation of incidence estimates for the population-based method is adequately described in the methods section as written**

Peer Review Comment: Population-based Method
The population-based method is clearly described and uses reliable population data. However, the changing PHO case definitions (Ab+ before 2009, RNA+ after 2018) may limit comparability across years.

**Reduced comparability of population-based method estimates due to changes in the HCV case definition is mentioned as a limitation in paragraph 3 of the discussion section. Additionally, a sensitivity analysis was carried out to investigate this non-comparability. Figure 4 plots population-based method using the PHO HCV case definition each year, using HCV Ab+ and using HCV RNA+. The results of this sensitivity analysis demonstrate the impact of changes in the HCV case definition on HCV incidence estimates for the population-based method.**

The authors should clarify how pre-existing infections were excluded from new cases and consider presenting 95% confidence intervals for annual incidence rates.

**Individuals included as HCV cases in a prior year were considered pre-existing infections and not included in the risk set for population-based method estimates in subsequent years. However, HCV testing data for Ontario was not available before 1999, so individuals that tested HCV+ prior to 1999 could not be identified as pre-existing HCV cases. The sensitivity analysis in which the index date was moved to 2004 was intended to explore how incidence estimates for each method are influenced by this left censoring of HCV testing data. This is now emphasised in the description of this sensitivity analysis:**

**“HCV testing data before January 1st, 1999 were unavailable for the present analysis. This may have affected HCV incidence estimates because follow-up eligibility for each individual depended on HCV test results in prior years. To evaluate the robustness of incidence estimates for each method to the unavailability of HCV testing data before 1999, we conducted a sensitivity analysis that estimated annual HCV incidence for each method after excluding HCV testing data from before 2004.”**

**Confidence intervals for incidence estimates are now reported in a supplemental table (S2 Table). Confidence bands were not reported in the HCV incidence figure for visual clarity.**

Peer Review Comment: The test-negative method
The Discussion section provides a good interpretation of the test-negative approach, but the Methods section should more clearly describe how individuals entered the cohort, how repeat testers were handled, and how infection dates were assigned. Adding these methodological details would enhance clarity and reproducibility.

**The methods section now explicitly defines the mechanism of cohort entry, modelling of repeat testing for the test-negative method:**

**“The test-negative method defined follow-up start as the date an individual tested HCV Ab- or HCV RNA-. Incident HCV infection was defined as: 1) testing HCV Ab+ or HCV RNA+ for those with a baseline HCV Ab- test result or 2) testing HCV RNA+ for those with a baseline HCV RNA- test result. The date an individual tested HCV+ was used as their date of infection. HCV+ individuals could re-enter the at-risk population if they subsequently tested HCV RNA-, indicating infection was cured or cleared. Follow-up for HCV-recovered individuals began on the date they tested HCV RNA-. In the case of individuals who tested HCV+ then HCV RNA- in the same year, their follow-up began on January 1^st^ of the following year.”**

Peer Review Comment: The RNA-based method
While the Discussion section clearly highlights the strengths of the RNA-based method, the Methods section should include more detail on how RNA test results were linked to antibody data and how unconfirmed Ab+ cases were handled in the analysis. Clarifying these steps would strengthen the methodological transparency of this approach.

**Antibody test results were not used in the RNA-based method, this is now mentioned more explicitly in the methods section:**

**“The RNA-based method used only HCV RNA test results and followed individuals that either: 1) never tested HCV RNA+ or 2) had cleared HCV infection (defined as an RNA+ test result followed by an RNA- test result).”**

Peer Review Comment: Antibody inclusive Method
The Discussion section effectively explains the rationale and limitations of the antibody-inclusive method, but the Methods section should briefly describe how Ab+ results were classified when follow-up RNA testing was missing, and how this approach avoided immortal-time bias. Including these methodological notes would improve clarity and reproducibility.

**The authors appreciate this comment. The methods section now elaborates further on how HCV Ab+ test results were used in the antibody-inclusive method and how the authors aimed to avoid immortal time bias.**

**“The antibody-inclusive method used the same cohort as the RNA-based method and defined incident HCV infection as the first recorded HCV Ab+ test for an individual, or an RNA+ test. A first-time antibody test was defined as an HCV Ab+ test result that was not preceded by an HCV RNA+ test result. First-time HCV Ab+ tests were used as cases regardless of whether they were confirmed by RNA testing. This was necessary because defining HCV Ab+ test results as cases conditional on the absence of subsequent RNA testing could introduce immortal time bias. As described above, annual HCV incidence-density was calculated using Poisson regression, and individuals could re-enter the risk set in a later year if they tested HCV RNA-.”**

Peer Review Summary Results and Discussion
All results and discussions are clear and directly relevant to the study content.
They present analytical steps and findings accurately and in full alignment with the text.
No major revisions are required.

Peer Review Comment Conclusion Section
A separate Conclusion section is missing.
Adding a short, clearly labeled paragraph summarizing key findings and public health implications would improve the paper’s structure.

**The manuscript now closes with the following explicitly labelled concluding paragraph**

**“Conclusion**

**The present study compared four methods for estimating the incidence of HCV using extant HCV testing data. The population-based method was efficient but more prone to misclassification than the test-negative method. The test-negative method, however, provided incidence estimates that may only be generalizable to high-risk populations. HCV incidence estimates were likely more generalizable for the RNA-based and antibody-inclusive methods. Unconfirmed HCV Ab+ tests led HCV incidence to be underestimated in the RNA-based method and overestimated in the antibody-inclusive method. These results provide much needed guidance for measuring progress towards WHO HCV incidence targets in regions that rely on extant HCV testing data.”**

Reviewer #2:

ScreenIT identified Randomization, Blinding, and Power analysis as potentially missing from the manuscript methods section.

**These methods do not apply to the present manuscript.**

ScreenIT: “antibody-inclusive suggested: None. Please add an RRID to help others identify

this resource”

**The laboratory methods used by PHO to measure HCV antibodies and HCV RNA are now discussed in the methods section. We provided as much detail as possible, although RRID’s were not provided in the PHO documentation.**

**“HCV Ab testing was carried out in PHO laboratories using chemiluminescent microparticle immunoassay and HCV RNA testing used the Roche cobas® 6800 system^1,2^**

1. **Ontario Agency for Health Protection and Promotion (Public Health Ontario). Hepatitis C – Diagnostic Serology 2024 [Available from:** [**https://www.publichealthontario.ca/en/Laboratory-Services/Test-Information-Index/Hepatitis-C-Diagnostic-Serology**](https://www.publichealthontario.ca/en/Laboratory-Services/Test-Information-Index/Hepatitis-C-Diagnostic-Serology)**.**
2. **Ontario Agency for Health Protection and Promotion (Public Health Ontario). Hepatitis C Virus RNA (HCV RNA) Testing and Interpretation Update. 2019.**

ScreenIT: “If you wrote code to analyze your data, please consider sharing the code in a public repository. This makes it easier for others to reproduce your analyses, and may also aid others seeking to analyze similar datasets.” And “If permitted, sharing data on a public repository with a persistent identifier (a DOI or accession number) can improve reproducibility and make it easier for other scientists to expand on your work”

**ICES data security and privacy prohibit the authors from making the data or the analytic code openly available, although they may be requested under certain conditions (as mentioned in the data availability statement in the manuscript).**

**“Data availability statement:The dataset from this study is held securely in coded form at ICES. While legal data sharing agreements between ICES and data providers (e.g., healthcare organizations and government) prohibit ICES from making the dataset publicly available, access may be granted to those who meet pre-specified criteria for confidential access, available at www.ices.on.ca/DAS (email: das@ices.on.ca). The full dataset creation plan and underlying analytic code may be available from the authors upon request, understanding that the computer programs may rely upon coding templates or macros that are unique to ICES and are therefore either inaccessible or may require modification. Analysis was carried out using SAS Enterprise Guide, Version 8.3 (SAS Institute Inc., Cary, NC, USA).”**

Reviewer #3: While the manuscript clearly explains the four methodological approaches and thoughtfully describes the advantages and disadvantages of each, the Discussion section would benefit from deeper consideration of their practical implications. The availability of real world testing data is a strength of the study, yet the authors do not fully illustrate how each method might perform when applied in high burden settings, where testing patterns, access to care, and reinfection risk differ substantially from low burden regions. Inclusion of concrete examples—such as regions where HCV incidence is driven by high community transmission, health care associated exposures, or concentrated epidemics—would help readers better understand the circumstances under which a given method is most reliable or feasible.

**The authors appreciate the Reviewer’s thoughtful comment. We agree that discussion of how these methods may perform outside of Canada will strengthen the relevance of this work in a global context. The following two paragraphs were added to the discussion section to elaborate on this topic:**

**“The utility of the methods discussed in the present study will likely vary across contexts depending on HCV burden, mode of infection, and HCV testing and reporting practices. These methods are not applicable in regions that do not have HCV surveillance systems that comprehensively record positive HCV test results, such as some low-middle income countries (LMIC.^1^ The generalizability of the test-negative method could potentially be higher in regions where universal HCV screening has been adopted, because individuals that test HCV- would likely better represent the underlying population rather than high-risk sub-groups. Universal HCV screening has been implemented in populations where medical procedures are a major mode of HCV transmission unlike Canada where HCV primarily spreads through high risk behaviors such as IDU.^2^ The test-negative method relies on cataloging of negative HCV test results: a report on HCV elimination efforts in high burden countries found that registries of HCV test results were only kept in 55% of these countries.^1^ In regions like the USA with HCV surveillance systems and where testing practices are shifting from risk-based to universal HCV screening, the test-negative method could become a valid approach for measuring HCV incidence.^3^**

**The performance of the RNA-based and antibody-inclusive methods will likely vary depending on the rate of unconfirmed HCV Ab+ test results, which is especially high in some settings where HCV RNA testing is not commonly available.^4^ The RNA-based method would likely perform poorly in these regions, making the antibody-inclusive method preferable. However, as point-of-care RNA testing is adopted in more regions, the applicability of the RNA-based method could improve.^1,5^ Additionally, HCV laboratory testing protocols in Ontario are moving towards reflexive HCV RNA testing on blood samples that are HCV Ab+, which will substantially decrease the rate of unconfirmed HCV Ab+ tests and improve the applicability of the RNA-based method.^6,7^”**

1. **Hiebert-Suwondo L, Manning J, Tohme RA, et al. A 2024 global report on national policies, programmes, and progress towards hepatitis C elimination: findings from 33 hepatitis elimination profiles. Lancet Gastroenterol Hepatol. Jul 2025;10(7):685-700. doi:10.1016/S2468-1253(25)00068-8**
2. **Stroffolini T, Stroffolini G. Prevalence and Modes of Transmission of Hepatitis C Virus Infection: A Historical Worldwide Review. Viruses. Jul 11 2024;16(7):1115-1132. doi:10.3390/v16071115**
3. **Schillie S, Wester C, Osborne M, Wesolowski L, Ryerson AB. CDC Recommendations for Hepatitis C Screening Among Adults - United States, 2020. MMWR Recomm Rep. Apr 10 2020;69(2):1-17. doi:10.15585/mmwr.rr6902a1**
4. **Graham CS, Swan T. A path to eradication of hepatitis C in low- and middle-income countries. Antiviral Res. Jul 2015;119:89-96. doi:10.1016/j.antiviral.2015.01.004**
5. **Trickey A, Fajardo E, Alemu D, Artenie AA, Easterbrook P. Impact of hepatitis C virus point-of-care RNA viral load testing compared with laboratory-based testing on uptake of RNA testing and treatment, and turnaround times: a systematic review and meta-analysis. Lancet Gastroenterol Hepatol. Mar 2023;8(3):253-270. doi:10.1016/S2468-1253(22)00346-6**
6. **Ontario Hepatitis C Elimination Planning Group ACaWG. The Ontario Hepatitis C Elimination Roadmap. 2023.**
7. **Ontario Agency for Health Protection and Promotion (Public Health Ontario). Hepatitis C – Diagnostic Serology. Accessed January 10th, 2026. https://www.publichealthontario.ca/en/Laboratory-Services/Test-Information-Index/Hepatitis-C-Diagnostic-Serology**

Additionally, the Discussion does not engage with the ongoing debate around incidence versus prevalence (or force of infection) as metrics for monitoring HCV elimination progress. Although prevalence has historically been used more widely in the HCV field, many high burden regions rely on incidence estimates derived from positive viral load results, a practice that is pragmatic and often accepted despite its limitations. A balanced argument acknowledging the strengths and limitations of incidence based monitoring—especially where repeat testing and case ascertainment vary—would strengthen the manuscript and increase its relevance to public health programs.

**The authors agree that these results should contribute to the discussion of how HCV burden and achievement of WHO HCV elimination targets should be measured in relation to data constraints. The following paragraphs were added to integrate our results with the existing work in this area.**

**“Reducing the global burden of HCV involves preventing new HCV infections (incident infections) and curing existing HCV infections (prevalent infections).^1^ HCV incidence** aims to measures **the rate of new HCV infections in a population over time, and is therefore a useful metric to measure the success of preventative interventions. Prospective cohort studies are the gold standard for measuring HCV incidence because this method differentiates incident and prevalent HCV infections with high accuracy.^2,3^ The use of extant HCV testing data is accepted by the WHO as a pragmatic alternative for measuring HCV incidence when prospective cohort studies are infeasible, with acknowledgement of important limitations.^2,4^ Extant testing data provides the date of HCV diagnosis and can be an imperfect proxy for the date of HCV infection if chronic HCV infection persists for years before diagnosis.^5^ While the date of HCV infection can be approximated using the duration between an HCV- test date and HCV+ test date (e.g., the midpoint), this method is imprecise when the mean duration between negative and positive tests is long (4 years in the present study).**

**The WHO strategy to reduce the global burden of HCV also emphasizes the need for programs that increase the rate of HCV screening and the availability and uptake of direct-acting antiviral therapy.^1^ The success of such programs is ideally measured using repeated estimates of HCV viremic prevalence, which is the proportion of individuals in a population that are HCV RNA+ at a point in time.^3^ Extant HCV testing data is frequently used to measure HCV prevalence, likely because HCV RNA+ test results can provide evidence of prevalent HCV infection.^6,7^ However, this method will underestimate HCV prevalence in regions such as Canada, where its estimated that 25% of chronic HCV infections are undiagnosed and therefore absent in repositories of extant testing data.^8^ Multiple approaches have been used in past studies to incorporate undiagnosed chronic HCV cases into estimates of HCV prevalence, such as back-calculation modelling and machine learning to impute undiagnosed HCV cases based on administrative health data.^8,9^”**

Journal Requirements:

When submitting your revision, we need you to address these additional requirements.

1. Please ensure that your manuscript meets PLOS ONE's style requirements, including those for file naming. The PLOS ONE style templates can be found at

<https://journals.plos.org/plosone/s/file?id=wjVg/PLOSOne_formatting_sample_main_body.pdf> and

[https://journals.plos.org/plosone/s/file?id=ba62/PLOSOne_formatting_sample_title_authors_affiliations.pdf](https://track.editorialmanager.com/CL0/https:%2F%2Fjournals.plos.org%2Fplosone%2Fs%2Ffile%3Fid=ba62%2FPLOSOne_formatting_sample_title_authors_affiliations.pdf/1/010f019ae24a4643-6cd9fef8-5c41-42b8-8146-7e8736d056d7-000000/O0LhkCpKQjKUnGN6-AnzAa4YQcPZH2Z1Lcfk9j0wZBQ=240).

**Manuscript formatting and file names now adhere to author guidelines**

2. Thank you for stating the following financial disclosure:

“Natural Sciences and Engineering Research Council of Canada Discovery grant and the Queen’s Fund for Scholarly work”

Please state what role the funders took in the study.  If the funders had no role, please state: "The funders had no role in study design, data collection and analysis, decision to publish, or preparation of the manuscript."

If this statement is not correct you must amend it as needed.

Please include this amended Role of Funder statement in your cover letter; we will change the online submission form on your behalf.

**The following statement now appears in the cover letter…**

**“This study was supported by ICES, which is funded by an annual grant from the Ontario Ministry of Health (MOH) and the Ministry of Long-Term Care (MLTC). This work was funded by the Natural Sciences and Engineering Research Council of Canada Discovery grant and the Queen’s Fund for Scholarly work. The funders had no role in study design, data collection and analysis, decision to publish, or preparation of the manuscript.”**

3. We note that you have indicated that there are restrictions to data sharing for this study. For studies involving human research participant data or other sensitive data, we encourage authors to share de-identified or anonymized data. However, when data cannot be publicly shared for ethical reasons, we allow authors to make their data sets available upon request. For information on unacceptable data access restrictions, please see <http://journals.plos.org/plosone/s/data-availability#loc-unacceptable-data-access-restrictions>.

Before we proceed with your manuscript, please address the following prompts:

a) If there are ethical or legal restrictions on sharing a de-identified data set, please explain them in detail (e.g., data contain potentially identifying or sensitive patient information, data are owned by a third-party organization, etc.) and who has imposed them (e.g., a Research Ethics Committee or Institutional Review Board, etc.). Please also provide contact information for a data access committee, ethics committee, or other institutional body to which data requests may be sent.

**Thank you for the opportunity to clarify the legal and ethical restrictions governing access to the dataset used in this study.**

**The data used in this study were accessed through ICES, an independent, non-profit research institute in Ontario, Canada. ICES is designated as a Prescribed Entity under Ontario’s Personal Health Information Protection Act (PHIPA). As such, ICES is legally authorized to collect and use personal health information for health system research without individual consent, provided that strict privacy and security requirements are met.**

**However, these same statutory provisions prohibit ICES from making record-level data publicly available. Specifically. Data are derived from provincial health administrative databases and contain personal health information and indirect identifiers. ICES’ authority to disclose identifiable or risk-reduced coded data is governed by PHIPA and related legislation (e.g., Coroners Act, CYFSA). Any disclosure of record-level data for research purposes requires Research Ethics Board (REB) approval, completion of a Privacy Impact Assessment (PIA), execution of a formal legal agreement, secure access within the ICES Data Environment, compliance with data minimization and risk management procedures.**

**These requirements are established under ICES’ Disclosure of ICES Data Policy (current version 2025-10-31), which outlines statutory obligations under PHIPA and associated regulations. As a result, ICES is legally prohibited from releasing the analytic dataset publicly. This restriction is imposed by provincial legislation and formal data sharing agreements between ICES and its data providers (including Ontario healthcare organizations and government agencies). Researchers who meet ICES’ eligibility criteria may request access to the data through ICES’ Data & Analytic Services (DAS) program. All applications undergo review to ensure compliance with applicable legislation and privacy requirements.**

**Data access requests may be directed to:**

**ICES Data & Analytic Services (DAS)
Website:** [**https://www.ices.on.ca/DAS**](https://www.ices.on.ca/DAS) **Email: das@ices.on.ca**

**The authors do not control access to the data and are not the sole gatekeepers. Interested researchers may apply to ICES directly through the formal DAS process. In addition, the dataset creation plan and analytic code used in this study are available from the corresponding author upon reasonable request, noting that some components may rely on ICES-specific macros or templates.**

**The full ICES data disclosure policy has been attached in the submission in the PDF titled “Disclosure of ICES Data Policy”**

4. Thank you for stating the following in the Acknowledgments Section of your manuscript:

“This study was supported by ICES, which is funded by an annual grant from the Ontario Ministry of Health (MOH) and the Ministry of Long-Term Care (MLTC). This document used data adapted from the Statistics Canada Postal Code^OM^ Conversion File, which is based on data licensed from Canada Post Corporation, and/or data adapted from the Ontario Ministry of Health Postal Code Conversion File, which contains data copied under license from ©Canada Post Corporation and Statistics Canada. Parts of this material are based on data and information compiled and provided by the Ontario Ministry of Health and Statistics Canada.”

We note that you have provided additional information within the Acknowledgements Section that is not currently declared in your Funding Statement. Please note that funding information should not appear in the Acknowledgments section or other areas of your manuscript. We will only publish funding information present in the Funding Statement section of the online submission form.

Please remove any funding-related text from the manuscript and let us know how you would like to update your Funding Statement. Currently, your Funding Statement reads as follows:

“Natural Sciences and Engineering Research Council of Canada Discovery grant and the Queen’s Fund for Scholarly work”

Please include your amended statements within your cover letter; we will change the online submission form on your behalf.

**Finding information has been removed from the acknowledgements section and added to the funding statement in the revised cover letter**

5. Please include captions for your Supporting Information files at the end of your manuscript, and update any in-text citations to match accordingly. Please see our Supporting Information guidelines for more information: <http://journals.plos.org/plosone/s/supporting-information>.

**Captions for supporting information files are now included at the end of the manuscript**

6. If the reviewer comments include a recommendation to cite specific previously published works, please review and evaluate these publications to determine whether they are relevant and should be cited. There is no requirement to cite these works unless the editor has indicated otherwise.

**Reviewer comments did not recommend citation of any specific works**

7. Please review your reference list to ensure that it is complete and correct. If you have cited papers that have been retracted, please include the rationale for doing so in the manuscript text, or remove these references and replace them with relevant current references. Any changes to the reference list should be mentioned in the rebuttal letter that accompanies your revised manuscript. If you need to cite a retracted article, indicate the article’s retracted status in the References list and also include a citation and full reference for the retraction notice.

**Several citations were added to the revised manuscript. This rebuttal letter outlines each new citation and the paragraph in which the citation was used**

1.	Ontario Agency for Health Protection and Promotion (Public Health Ontario). Hepatitis C – Diagnostic Serology. Accessed January 10th, 2026. <https://www.publichealthontario.ca/en/Laboratory-Services/Test-Information-Index/Hepatitis-C-Diagnostic-Serology>
