# Supplementary material for: Four methods for estimating hepatitis C incidence using extant testing data
Source: PLoS One. 2026 Jun 10;21(6):e0335115. doi: 10.1371/journal.pone.0335115 (PMC13252766; doi:10.1371/journal.pone.0335115)
Supplement: Attachment — Submitted filename: Response to Reviewers.docx [file pone.0335115.s008.docx]

Round 2 Response to Reviewers

Reviewer #1: Dear Editor,
The manuscript has improved well and the authors addressed the comments.
I recommend acceptance after minor revision.
Minor points:

1. Confidence intervals (95% CI) should be included in Figure 3 (e.g., by adding error bars) to improve clarity and interpretation of the results.

**Figure 3 now contains error bars representing the 95% confidence interval of each annual estimate, and this is reflected in the figure description. Point markers (e.g., circle, square, triangle) were removed for better visualization of the confidence interval bars.**

2. Clarify the implications of the imprecision in the midpoint approach, particularly how it may affect the timing and accuracy of incidence estimates. For example, explain whether this approach could lead to misclassification of infection timing, distortion of temporal trends, or over- or underestimation of incidence rates when the interval between tests is long.

**These points are now discussed in paragraph 6 of the discussion section:**

**“The test-negative method had the advantage of being able to assign the date of HCV infection to be the midpoint between HCV- and HCV+ test dates. This method has been used in previous studies to improve precision in the classification of HCV infection date by assuming that the average date of infection occurred halfway between negative and positive HCV test dates.[11, 24] While the true date of infection must fall in this window, choosing the midpoint to represent infection date risks misclassification of HCV infection date, especially when the duration between the negative and positive test is long (average of 4 years in the present study). This change inflated HCV incidence estimates earlier in follow-up and diminished them later in follow-up (Fig 5). This is because cases were only reassigned from later years to earlier years; 1999 gained HCV cases while losing none, and the ratio of lost cases to gained cases increased for each year of follow-up until 2018 when HCV cases were only lost. It is therefore important to recognize that when HCV testing data is only available for a specific window of time, reassigning infection dates to the midpoint between an HCV- and HCV+ test could lead to the potentially false conclusion that the incidence of HCV is decreasing.”**

3. Provide a more structured comparison of the strengths and limitations of the different methods.

**In the previous submission, the performance of each method was discussed systematically one at a time, without mention of the other methods. In the revised submission, listed strengths and limitations for a given method now also include discussion of how the other methods fare with respect to the strength or limitation in question. This gives structure to the comparison of methods without reorganizing the flow of information in the discussion section.**
