# Supplementary material for: Four methods for estimating hepatitis C incidence using extant testing data
Source: PLoS One. 2026 Jun 10;21(6):e0335115. doi: 10.1371/journal.pone.0335115 (PMC13252766; doi:10.1371/journal.pone.0335115)
Supplement: Peer review history

# Peer review: Decision Letter 0

**DOI:** 10.1371/journal.pone.0335115.r001

**Authors:** Hamid Sharifi

2 Dec 2025

PONE-D-25-54063

Four methods for estimating hepatitis C incidence using extant testing data

PLOS ONE

Dear Dr. McFarlane,

Thank you for submitting your manuscript to PLOS ONE. After careful consideration, we feel that it has merit but does not fully meet PLOS ONE’s publication criteria as it currently stands. Therefore, we invite you to submit a revised version of the manuscript that addresses the points raised during the review process.

Please submit your revised manuscript by Jan 16 2026 11:59PM. If you will need more time than this to complete your revisions, please reply to this message or contact the journal office at plosone@plos.org. When you're ready to submit your revision, log on to https://www.editorialmanager.com/pone/ and select the 'Submissions Needing Revision' folder to locate your manuscript file.

Please include the following items when submitting your revised manuscript:

- A rebuttal letter that responds to each point raised by the academic editor and reviewer(s). You should upload this letter as a separate file labeled 'Response to Reviewers'.
- A marked-up copy of your manuscript that highlights changes made to the original version. You should upload this as a separate file labeled 'Revised Manuscript with Track Changes'.
- An unmarked version of your revised paper without tracked changes. You should upload this as a separate file labeled 'Manuscript'.

-->If you would like to make changes to your financial disclosure, please include your updated statement in your cover letter. Guidelines for resubmitting your figure files are available below the reviewer comments at the end of this letter.

If applicable, we recommend that you deposit your laboratory protocols in protocols.io to enhance the reproducibility of your results. Protocols.io assigns your protocol its own identifier (DOI) so that it can be cited independently in the future. For instructions see: https://journals.plos.org/plosone/s/submission-guidelines#loc-laboratory-protocols. Additionally, PLOS ONE offers an option for publishing peer-reviewed Lab Protocol articles, which describe protocols hosted on protocols.io. Read more information on sharing protocols at https://plos.org/protocols?utm_medium=editorial-email&utm_source=authorletters&utm_campaign=protocols.

We look forward to receiving your revised manuscript.

Kind regards,

Hamid Sharifi, Professor

Academic Editor

PLOS ONE

Journal Requirements:

When submitting your revision, we need you to address these additional requirements.

1. Please ensure that your manuscript meets PLOS ONE's style requirements, including those for file naming. The PLOS ONE style templates can be found at

https://journals.plos.org/plosone/s/file?id=wjVg/PLOSOne_formatting_sample_main_body.pdf and

https://journals.plos.org/plosone/s/file?id=ba62/PLOSOne_formatting_sample_title_authors_affiliations.pdf.

2. Thank you for stating the following financial disclosure:

“Natural Sciences and Engineering Research Council of Canada Discovery grant and the Queen’s Fund for Scholarly work”

Please state what role the funders took in the study.  If the funders had no role, please state: "The funders had no role in study design, data collection and analysis, decision to publish, or preparation of the manuscript."

If this statement is not correct you must amend it as needed.

Please include this amended Role of Funder statement in your cover letter; we will change the online submission form on your behalf.

3. We note that you have indicated that there are restrictions to data sharing for this study. For studies involving human research participant data or other sensitive data, we encourage authors to share de-identified or anonymized data. However, when data cannot be publicly shared for ethical reasons, we allow authors to make their data sets available upon request. For information on unacceptable data access restrictions, please see http://journals.plos.org/plosone/s/data-availability#loc-unacceptable-data-access-restrictions.

Before we proceed with your manuscript, please address the following prompts:

a) If there are ethical or legal restrictions on sharing a de-identified data set, please explain them in detail (e.g., data contain potentially identifying or sensitive patient information, data are owned by a third-party organization, etc.) and who has imposed them (e.g., a Research Ethics Committee or Institutional Review Board, etc.). Please also provide contact information for a data access committee, ethics committee, or other institutional body to which data requests may be sent.

b) If there are no restrictions, please upload the minimal anonymized data set necessary to replicate your study findings to a stable, public repository and provide us with the relevant URLs, DOIs, or accession numbers. Please see http://www.bmj.com/content/340/bmj.c181.long for guidelines on how to de-identify and prepare clinical data for publication. For a list of recommended repositories, please see https://journals.plos.org/plosone/s/recommended-repositories. You also have the option of uploading the data as Supporting Information files, but we would recommend depositing data directly to a data repository if possible.

Please update your Data Availability statement in the submission form accordingly.

4. Thank you for stating the following in the Acknowledgments Section of your manuscript:

“This study was supported by ICES, which is funded by an annual grant from the Ontario Ministry of Health (MOH) and the Ministry of Long-Term Care (MLTC). This document used data adapted from the Statistics Canada Postal Code^OM^ Conversion File, which is based on data licensed from Canada Post Corporation, and/or data adapted from the Ontario Ministry of Health Postal Code Conversion File, which contains data copied under license from ©Canada Post Corporation and Statistics Canada. Parts of this material are based on data and information compiled and provided by the Ontario Ministry of Health and Statistics Canada.”

We note that you have provided additional information within the Acknowledgements Section that is not currently declared in your Funding Statement. Please note that funding information should not appear in the Acknowledgments section or other areas of your manuscript. We will only publish funding information present in the Funding Statement section of the online submission form.

Please remove any funding-related text from the manuscript and let us know how you would like to update your Funding Statement. Currently, your Funding Statement reads as follows:

“Natural Sciences and Engineering Research Council of Canada Discovery grant and the Queen’s Fund for Scholarly work”

Please include your amended statements within your cover letter; we will change the online submission form on your behalf.

5. Please include captions for your Supporting Information files at the end of your manuscript, and update any in-text citations to match accordingly. Please see our Supporting Information guidelines for more information: http://journals.plos.org/plosone/s/supporting-information.

6. If the reviewer comments include a recommendation to cite specific previously published works, please review and evaluate these publications to determine whether they are relevant and should be cited. There is no requirement to cite these works unless the editor has indicated otherwise.

7. Please review your reference list to ensure that it is complete and correct. If you have cited papers that have been retracted, please include the rationale for doing so in the manuscript text, or remove these references and replace them with relevant current references. Any changes to the reference list should be mentioned in the rebuttal letter that accompanies your revised manuscript. If you need to cite a retracted article, indicate the article’s retracted status in the References list and also include a citation and full reference for the retraction notice.

[Note: HTML markup is below. Please do not edit.]

Reviewers' comments:

Reviewer's Responses to Questions

**Comments to the Author**

1. Is the manuscript technically sound, and do the data support the conclusions?

The manuscript must describe a technically sound piece of scientific research with data that supports the conclusions. Experiments must have been conducted rigorously, with appropriate controls, replication, and sample sizes. The conclusions must be drawn appropriately based on the data presented.

Reviewer #1: Yes

Reviewer #2: Yes

Reviewer #3: Yes

**********

2. Has the statistical analysis been performed appropriately and rigorously?

Reviewer #1: Yes

Reviewer #2: Yes

Reviewer #3: Yes

**********

3. Have the authors made all data underlying the findings in their manuscript fully available?

The PLOS Data policy requires authors to make all data underlying the findings described in their manuscript fully available without restriction, with rare exception (please refer to the Data Availability Statement in the manuscript PDF file). The data should be provided as part of the manuscript or its supporting information, or deposited to a public repository. For example, in addition to summary statistics, the data points behind means, medians and variance measures should be available. If there are restrictions on publicly sharing data—e.g. participant privacy or use of data from a third party—those must be specified.

Reviewer #1: Yes

Reviewer #2: Yes

Reviewer #3: Yes

**********

4. Is the manuscript presented in an intelligible fashion and written in standard English?

PLOS ONE does not copyedit accepted manuscripts, so the language in submitted articles must be clear, correct, and unambiguous. Any typographical or grammatical errors should be corrected at revision, so please note any specific errors here.

Reviewer #1: Yes

Reviewer #2: Yes

Reviewer #3: Yes

**********

5. Review Comments to the Author

Please use the space provided to explain your answers to the questions above. You may also include additional comments for the author, including concerns about dual publication, research ethics, or publication ethics. (Please upload your review as an attachment if it exceeds 20,000 characters)

Reviewer #1: Four methods for estimating hepatitis C incidence using extant testing data

Dear Authors,

Below are detailed comments organized by manuscript section to guide your revisions and enhance clarity, transparency, and reproducibility.

The abstract is well structured and summarizes the study clearly.

However, several improvements would strengthen its clarity and scientific impact:

1. Background: Add one introductory sentence linking HCV incidence estimation to its global public health relevance.

2. Methods: Clarify the data source (ICES, Ontario) and briefly mention the statistical method used (e.g., Poisson regression).

3. Results: Use a short opening phrase (e.g., Results showed distinct incidence patterns across methods) and simplify wording such as influenced by → sensitive to.

4. Conclusion: Strengthen the final statement by linking it to public health applications or surveillance implications.

Peer Review Introduction Section

1. Add a recent statistic on global HCV prevalence to strengthen the topic’s importance.

2. Include a practical example of a health intervention that relies on incidence estimation.

3. Briefly hint at the types of strengths and limitations of the methods.

4. Add a concluding sentence explaining how the comparison can improve public health policies.

Peer Review Comment Section: Data Source

Data sources are clearly presented and well-linked across databases. Unique identifiers prevent duplication, and coverage from all 11 PHO labs is a key strength. Minor clarifications are needed regarding duplication checks, data validation, and potential selection bias.

Peer Review Comment Section: HCV Incidence Estimation Methods

Although the Results and Discussion describe the four methods well, the Methods section still needs a brief explanation of how incidence was calculated for each approach and how comparability was ensured. Adding these details would improve methodological clarity and reproducibility.

Peer Review Comment: Population-based Method

The population-based method is clearly described and uses reliable population data. However, the changing PHO case definitions (Ab+ before 2009, RNA+ after 2018) may limit comparability across years. The authors should clarify how pre-existing infections were excluded from new cases and consider presenting 95% confidence intervals for annual incidence rates.

Peer Review Comment: The test-negative method

The Discussion section provides a good interpretation of the test-negative approach, but the Methods section should more clearly describe how individuals entered the cohort, how repeat testers were handled, and how infection dates were assigned. Adding these methodological details would enhance clarity and reproducibility.

Peer Review Comment: The RNA-based method

While the Discussion section clearly highlights the strengths of the RNA-based method, the Methods section should include more detail on how RNA test results were linked to antibody data and how unconfirmed Ab+ cases were handled in the analysis. Clarifying these steps would strengthen the methodological transparency of this approach.

Peer Review Comment: Antibody inclusive Method

The Discussion section effectively explains the rationale and limitations of the antibody-inclusive method, but the Methods section should briefly describe how Ab+ results were classified when follow-up RNA testing was missing, and how this approach avoided immortal-time bias.

Including these methodological notes would improve clarity and reproducibility.

Peer Review Summary Results and Discussion

All results and discussions are clear and directly relevant to the study content.

They present analytical steps and findings accurately and in full alignment with the text.

No major revisions are required.

Peer Review Comment Conclusion Section

A separate Conclusion section is missing.

Adding a short, clearly labeled paragraph summarizing key findings and public health implications would improve the paper’s structure.

Reviewed by: Dr. Abdulelah Mansoor Al-Ganad

Peer Reviewer, Epidemiology and Biostatistics

Reviewer #2: This is an automated report for PONE-D-25-54063. This report was solicited by the PLOS One editorial team and provided by ScreenIT.

ScreenIT is an independent group of scientists developing automated tools that analyze academic papers. A set of automated tools screened your submitted manuscript and provided the report below. Each tool was created by your academic colleagues with the goal of helping authors. The tools look for factors that are important for transparency, rigor and reproducibility, and we hope that the report might help you to improve reporting in your manuscript. Within the report you will find links to more information about the items that the tools check. These links include helpful papers, websites, or videos that explain why the item is important. While our screening tools aim to improve and maintain quality standards they may, on occasion, miss nuances specific to your study type or flag something incorrectly. Each tool has limitations that are described on the ScreenIT website. The tools screen the main file for the paper; they are not able to screen supplements stored in separate files. Please note that the Academic Editor had access to these comments while making a decision on your manuscript. The Academic Editor may ask that issues flagged in this report be addressed. If you would like to provide feedback on the ScreenIT tool, please email the team at ScreenIt@bih-charite.de. If you have questions or concerns about the review process, please contact the PLOS One office at plosone@plos.org.

Reviewer #3: While the manuscript clearly explains the four methodological approaches and thoughtfully describes the advantages and disadvantages of each, the Discussion section would benefit from deeper consideration of their practical implications. The availability of real‑world testing data is a strength of the study, yet the authors do not fully illustrate how each method might perform when applied in high‑burden settings, where testing patterns, access to care, and reinfection risk differ substantially from low‑burden regions. Inclusion of concrete examples—such as regions where HCV incidence is driven by high community transmission, health‑care associated exposures, or concentrated epidemics—would help readers better understand the circumstances under which a given method is most reliable or feasible.

Additionally, the Discussion does not engage with the ongoing debate around incidence versus prevalence (or force of infection) as metrics for monitoring HCV elimination progress. Although prevalence has historically been used more widely in the HCV field, many high‑burden regions rely on incidence estimates derived from positive viral load results, a practice that is pragmatic and often accepted despite its limitations. A balanced argument acknowledging the strengths and limitations of incidence‑based monitoring—especially where repeat testing and case ascertainment vary—would strengthen the manuscript and increase its relevance to public health programs.

**********

6. PLOS authors have the option to publish the peer review history of their article (what does this mean?). If published, this will include your full peer review and any attached files.

If you choose “no”, your identity will remain anonymous but your review may still be made public.

**Do you want your identity to be public for this peer review?** For information about this choice, including consent withdrawal, please see our Privacy Policy.

Reviewer #1: No

Reviewer #2: No

Reviewer #3: **Yes:** Aslam Muhammad Khawar

**********

[NOTE: If reviewer comments were submitted as an attachment file, they will be attached to this email and accessible via the submission site. Please log into your account, locate the manuscript record, and check for the action link "View Attachments". If this link does not appear, there are no attachment files.]

To ensure your figures meet our technical requirements, please review our figure guidelines: https://journals.plos.org/plosone/s/figures

You may also use PLOS’s free figure tool, NAAS, to help you prepare publication quality figures: https://journals.plos.org/plosone/s/figures#loc-tools-for-figure-preparation.

NAAS will assess whether your figures meet our technical requirements by comparing each figure against our figure specifications.

Attachment Submitted filename: report_10.1101+2025.10.08.25337617.pdf

# Author comment: Author response to Decision Letter 1

**DOI:** 10.1371/journal.pone.0335115.r002

26 Feb 2026

Reviewer Responses - Four methods for estimating hepatitis C incidence using extant testing data

Reviewer #1: Four methods for estimating hepatitis C incidence using extant testing data

Dear Authors,

Below are detailed comments organized by manuscript section to guide your revisions and enhance clarity, transparency, and reproducibility.

The abstract is well structured and summarizes the study clearly.

However, several improvements would strengthen its clarity and scientific impact:

1. Background: Add one introductory sentence linking HCV incidence estimation to its global public health relevance.

Thank you for this comment, relevance of this work is now framed in the context of HCV elimination goals set by the World Health Organization. This framework was chosen to align with previous publications investigating measurement of HCV incidence.1-3

“Accurate estimation of HCV incidence is crucial for measuring progress towards HCV elimination targets set by the World Health Organization (WHO).”

1. Mitchell HD, Clancy I, De Angelis D, et al. Using modelling and public health surveillance data to evidence progress towards the WHO incidence target for hepatitis C elimination in people who inject drugs - a review of complementary approaches in the United Kingdom. Int J Drug Policy. Dec 2025;146:105059. doi:10.1016/j.drugpo.2025.105059

2. Artenie A, Luhmann N, Lim AG, et al. Methods and indicators to validate country reductions in incidence of hepatitis C virus infection to elimination levels set by WHO. Lancet Gastroenterol Hepatol. Apr 2022;7(4):353-366. doi:10.1016/S2468-1253(21)00311-3

3. Popovic N, Williams A, Perinet S, et al. National Hepatitis C estimates: Incidence, prevalence, undiagnosed proportion and treatment, Canada, 2019. Can Commun Dis Rep. Nov 3 2022;48(11-12):540-549. doi:10.14745/ccdr.v48i1112a07

2. Methods: Clarify the data source (ICES, Ontario) and briefly mention the statistical method used (e.g., Poisson regression).

ICES is an independent, non-profit research institute whose legal status under Ontario’s health information privacy law allows it to collect and analyze health care and demographic data, without consent, for health system evaluation and improvement.

This is now reflected more clearly in the abstract:

“Methods: Using databases linked at ICES ¬– a repository of administrative health data for Ontario residents – we constructed a cohort of 15.8 million Ontarians aged 18–80 between 1999 and 2018 to estimate annual HCV incidence using four methods”

The methods section of the abstract now emphasizes that the population-based method used simple calculations to estimate HCV incidence, while the other three methods used Poisson regression.

“The population-based method calculated HCV incidence as the number of HCV cases confirmed through HCV testing each year divided by population size estimates, while Poisson regression was used in the other three incidence estimation methods”

3. Results: Use a short opening phrase (e.g., Results showed distinct incidence patterns across methods) and simplify wording such as influenced by → sensitive to.

Incidence estimates from each method in the results section of the abstract are now prefaced by the following statement:

“Distinct patterns of HCV incidence were found across the estimation methods…”

The following change was made:

“Population-based estimates were sensitive to changes in…”

4. Conclusion: Strengthen the final statement by linking it to public health applications or surveillance implications.

The concluding statement ties the significance of the results back into the evaluation of achievement of WHO HCV incidence targets”

“These new findings illustrate the influence of cohort definition and HCV case definition when estimating HCV incidence using extant testing data, which will support accurate measurement of progress towards WHO HCV elimination goals.”

Peer Review Introduction Section

1. Add a recent statistic on global HCV prevalence to strengthen the topic’s importance.

The introduction now opens with the following statistics:

“Approximately 242,000 individuals died from hepatitis C (HCV) infection globally in 2022 and around one million new HCV cases occur each year.1”

1. The World Health Organization. (2025, 25 July 2025). Hepatitis C. Retrieved January 22 from https://www.who.int/news-room/fact-sheets/detail/hepatitis-c

2. Include a practical example of a health intervention that relies on incidence estimation.

The authors agree that early discussion of the practical importance of HCV incidence estimation would strengthen the introduction. The following sentences were added to emphasize this importance and reference existing work in this area:

“Updated global HCV elimination targets were announced by the WHO in 2023, which included a reduction in HCV incidence to ≤5 new annual HCV cases per 100,000 adults by 2030.1 Since the quantity and quality of HCV testing data varies across regions, several approaches have been proposed for measuring progress towards this HCV incidence target.1-4”

1. The World Health Organization. Guidance for country validation of viral hepatitis elimination and path to elimination. Global HIV HaSPH; 2023. https://www.who.int/publications/i/item/9789240078635

2. The World Health Organization. Interim guidance for country validation of viral hepatitis elimination. Global HIV HaSPH; 2021. https://www.who.int/publications/i/item/9789240028395

3. Mitchell HD, Clancy I, De Angelis D, et al. Using modelling and public health surveillance data to evidence progress towards the WHO incidence target for hepatitis C elimination in people who inject drugs - a review of complementary approaches in the United Kingdom. Int J Drug Policy. Dec 2025;146:105059. doi:10.1016/j.drugpo.2025.105059

4. Artenie A, Luhmann N, Lim AG, et al. Methods and indicators to validate country reductions in incidence of hepatitis C virus infection to elimination levels set by WHO. Lancet Gastroenterol Hepatol. Apr 2022;7(4):353-366. doi:10.1016/S2468-1253(21)00311-3

3. Briefly hint at the types of strengths and limitations of the methods.

We appreciate the Reviewer’s comment, and would like to note that the strengths and limitations of each method are described in the introduction:

Paragraph 3 mentions that the population-based method is efficient but prone to misclassification, and that the test negative method is less prone to misclassification, possibly at the cost of generalizability.

Paragraph 4 mentions that the RNA-based method likely improves generalizability may underestimate HCV incidence due to unconfirmed Ab+ tests

Paragraph 5 mentions that the antibody-inclusive method makes use of unconfirmed Ab+ tests but may overestimate HCV incidence

4. Add a concluding sentence explaining how the comparison can improve public health policies.

The introduction now includes a concluding paragraph to reiterate that many regions rely on extant HCV testing data to measure progress towards HCV elimination goals, without necessarily understanding the limitations of this data source:

“When prospective cohort studies are infeasible, regions often rely on extant HCV testing data to measure progress towards WHO HCV elimination targets.1-3 HCV incidence can be measured using extant HCV testing data through a variety of approaches, the strengths and limitations of which are poorly understood. Therefore, the objective of this study was to use extant HCV testing data to estimate the annual incidence of HCV from 1999 to 2018 in Ontario using four different methods, and comment on the generalizability and potential biases for each method.”

1. The World Health Organization. Interim guidance for country validation of viral hepatitis elimination. Global HIV HaSPH; 2021. https://www.who.int/publications/i/item/9789240028395

2. The World Health Organization. Guidance for country validation of viral hepatitis elimination and path to elimination. Global HIV HaSPH; 2023. https://www.who.int/publications/i/item/9789240078635

3. Artenie A, Luhmann N, Lim AG, et al. Methods and indicators to validate country reductions in incidence of hepatitis C virus infection to elimination levels set by WHO. Lancet Gastroenterol Hepatol. Apr 2022;7(4):353-366. doi:10.1016/S2468-1253(21)00311-3

Peer Review Comment Section: Data Source

Data sources are clearly presented and well-linked across databases. Unique identifiers prevent duplication, and coverage from all 11 PHO labs is a key strength. Minor clarifications are needed regarding duplication checks, data validation, and potential selection bias.

The following statement was added to provide clarity on how duplicate tests were defined, and how specific duplicate test scenarios were handled at the data cleaning stage:

“HCV tests with the same individual identifier code and test date were considered duplicates. When duplicate records differed by test type, RNA tests were prioritized over antibody tests because RNA testing can provide definitive evidence of current/active infection. When multiple tests of the same type were recorded on the same day, positive test results were retained, as these were assumed to represent corrections of errors during laboratory analysis.”

Gaps in ascertainment of HCV testing data are now discussed in the following paragraph

"Private laboratories are authorized to carry out HCV Ab screening, but are not required to submit HCV Ab- test results to PHO.1 The absence of HCV Ab- test results from private laboratories in the present study could have reduced the denominator of the test-negative method cohort. Additionally, the absence of these test results could impact generalizability of results to individuals receiving HCV testing in private laboratories."

1. Ontario Agency for Health Protection and Promotion (Public Health Ontario). Hepatitis C – Diagnostic Serology. Accessed January 10th, 2026. https://www.publichealthontario.ca/en/Laboratory-Services/Test-Information-Index/Hepatitis-C-Diagnostic-Serology

Peer Review Comment Section: HCV Incidence Estimation Methods

Although the Results and Discussion describe the four methods well, the Methods section still needs a brief explanation of how incidence was calculated for each approach and how comparability was ensured. Adding these details would improve methodological clarity and reproducibility.

The following paragraph was added to the description of the test-negative method to clarify how HCV incidence was estimated

“Poisson regression with no covariates was used to model HCV diagnosis events for each individual as a function of follow-up time since their most recent negative test date, providing estimates of annual HCV incidence-density (in units of cases per 100,000 person-years).”

A brief statement was added after the RNA-based and antibody-inclusive methods to indicate that incidence estimation using Poisson regression was consistently used in these three methods:

“As described above, annual HCV incidence density was calculated using Poisson regression, and individuals could re-enter the risk set in a later year if they tested HCV RNA-.”

The manual calculation of incidence estimates for the population-based method is adequately described in the methods section as written

Peer Review Comment: Population-based Method

The population-based method is clearly described and uses reliable population data. However, the changing PHO case definitions (Ab+ before 2009, RNA+ after 2018) may limit comparability across years.

Reduced comparability of population-based method estimates due to changes in the HCV case definition is mentioned as a limitation in paragraph 3 of the discussion section. Additionally, a sensitivity analysis was carried out to investigate this non-comparability. Figure 4 plots population-based method using the PHO HCV case definition each year, using HCV Ab+ and using HCV RNA+. The results of this sensitivity analysis demonstrate the impact of changes in the HCV case definition on HCV incidence estimates for the population-based method.

The authors should clarify how pre-existing infections were excluded from new cases and consider presenting 95% confidence intervals for annual incidence rates.

Individuals included as HCV cases in a prior year were considered pre-existing infections and not included in the risk set for population-based method estimates in subsequent years. However, HCV testing data for Ontario was not available before 1999, so individuals that tested HCV+ prior to 1999 could not be identified as pre-existing HCV cases. The sensitivity analysis in which the index date was moved to 2004 was intended to explore how incidence estimates for each method are influenced by this left censoring of HCV testing data. This is now emphasised in the description of this sensitivity analysis:

“HCV testing data before January 1st, 1999 were unavailable for the present analysis. This may have affected HCV incidence estimates because follow-up eligibility for each individual depended on HCV test results in prior years. To evaluate the robustness of incidence estimates for each method to the unavailability of HCV testing data before 1999, we conducted a sensitivity analysis that estimated annual HCV incidence for each method after excluding HCV testing data from before 2004.”

Confidence intervals for incidence estimates are now reported in a supplemental table (S2 Table). Confidence bands were not reported in the HCV incidence figure for visual clarity.

Peer Review Comment: The test-negative method

The Discussion section provides a good interpretation of the test-negative approach, but the Methods section should more clearly describe how individuals entered the cohort, how repeat testers were handled, and how infection dates were assigned. Adding these methodological details would enhance clarity and reproducibility.

The methods section now explicitly defines the mechanism of cohort entry, modelling of repeat testing for the test-negative method:

“The test-negative method defined follow-up start as the date an individual tested HCV Ab- or HCV RNA-. Incident HCV infection was defined as: 1) testing HCV Ab+ or HCV RNA+ for those with a baseline HCV Ab- test result or 2) testing HCV RNA+ for those with a baseline HCV RNA- test result. The date an individual tested HCV+ was used as their date of infection. HCV+ individuals could re-enter the at-risk population if they subsequently tested HCV RNA-, indicating infection was cured or cleared. Follow-up for HCV-recovered individuals began on the date they tested HCV RNA-. In the case of individuals who tested HCV+ then HCV RNA- in the same year, their follow-up began on January 1st of the following year.”

Peer Review Comment: The RNA-based method

While the Discussion section clearly highlights the strengths of the RNA-based method, the Methods section should include more detail on how RNA test results were linked to antibody data and how unconfirmed Ab+ cases were handled in the analysis. Clarifying these steps would strengthen the methodological transparency of this approach.

Antibody test results were not used in the RNA-based method, this is now mentioned more explicitly in the methods section:

“The RNA-based method used only HCV RNA test results and followed individuals that either: 1) never tested HCV RNA+ or 2) had cleared HCV infection (defined as an RNA+ test result followed by an RNA- test result).”

Peer Review Comment: Antibody inclusive Method

The Discussion section effectively explains the rationale and limitations of the antibody-inclusive method, but the Methods section should briefly describe how Ab+ results were classified when follow-up RNA testing was missing, and how this approach avoided immortal-time bias. Including these methodological notes would improve clarity and reproducibility.

The authors appreciate this comment. The methods section now elaborates further on how HCV Ab+ test results were used in the antibody-inclusive method and how the authors aimed to avoid immortal time bias.

“The antibody-inclusive method used the same cohort as the RNA-based method and defined incident HCV infection as the first recorded HCV Ab+ test for an in

Attachment Submitted filename: Reviewer responses.docx

# Peer review: Decision Letter 1

**DOI:** 10.1371/journal.pone.0335115.r003

**Authors:** Hamid Sharifi

16 Apr 2026

PONE-D-25-54063R1

Four methods for estimating hepatitis C incidence using extant testing data

PLOS One

Dear Dr. McFarlane,

Thank you for submitting your manuscript to PLOS ONE. After careful consideration, we feel that it has merit but does not fully meet PLOS ONE’s publication criteria as it currently stands. Therefore, we invite you to submit a revised version of the manuscript that addresses the points raised during the review process.

Please submit your revised manuscript by May 31 2026 11:59PM. If you will need more time than this to complete your revisions, please reply to this message or contact the journal office at plosone@plos.org. When you're ready to submit your revision, log on to https://www.editorialmanager.com/pone/ and select the 'Submissions Needing Revision' folder to locate your manuscript file.

Please include the following items when submitting your revised manuscript:

- A letter that responds to each point raised by the academic editor and reviewer(s). You should upload this letter as a separate file labeled 'Response to Reviewers'.
- A marked-up copy of your manuscript that highlights changes made to the original version. You should upload this as a separate file labeled 'Revised Manuscript with Track Changes'.
- An unmarked version of your revised paper without tracked changes. You should upload this as a separate file labeled 'Manuscript'.

If you would like to make changes to your financial disclosure, please include your updated statement in your cover letter. Guidelines for resubmitting your figure files are available below the reviewer comments at the end of this letter.

If applicable, we recommend that you deposit your laboratory protocols in protocols.io to enhance the reproducibility of your results. Protocols.io assigns your protocol its own identifier (DOI) so that it can be cited independently in the future. For instructions see: https://journals.plos.org/plosone/s/submission-guidelines#loc-laboratory-protocols. Additionally, PLOS ONE offers an option for publishing peer-reviewed Lab Protocol articles, which describe protocols hosted on protocols.io. Read more information on sharing protocols at https://plos.org/protocols?utm_medium=editorial-email&utm_source=authorletters&utm_campaign=protocols.

As the corresponding author, your ORCID iD is verified in the submission system and will appear in the published article. PLOS supports the use of ORCID, and we encourage all coauthors to register for an ORCID iD and use it as well. Please encourage your coauthors to verify their ORCID iD within the submission system before final acceptance, as unverified ORCID iDs will not appear in the published article. *Only* the individual author can complete the verification step; PLOS staff *cannot* verify ORCID iDs on behalf of authors.

We look forward to receiving your revised manuscript.

Kind regards,

Hamid Sharifi, Professor

Academic Editor

PLOS One

Journal Requirements:

If the reviewer comments include a recommendation to cite specific previously published works, please review and evaluate these publications to determine whether they are relevant and should be cited. There is no requirement to cite these works unless the editor has indicated otherwise.

Please review your reference list to ensure that it is complete and correct. If you have cited papers that have been retracted, please include the rationale for doing so in the manuscript text, or remove these references and replace them with relevant current references. Any changes to the reference list should be mentioned in the rebuttal letter that accompanies your revised manuscript. If you need to cite a retracted article, indicate the article’s retracted status in the References list and also include a citation and full reference for the retraction notice.

[Note: HTML markup is below. Please do not edit.]

Reviewers' comments:

Reviewer's Responses to Questions

**Comments to the Author**

1. If the authors have adequately addressed your comments raised in a previous round of review and you feel that this manuscript is now acceptable for publication, you may indicate that here to bypass the “Comments to the Author” section, enter your conflict of interest statement in the “Confidential to Editor” section, and submit your "Accept" recommendation.

Reviewer #1: All comments have been addressed

Reviewer #3: All comments have been addressed

**********

2. Is the manuscript technically sound, and do the data support the conclusions?

The manuscript must describe a technically sound piece of scientific research with data that supports the conclusions. Experiments must have been conducted rigorously, with appropriate controls, replication, and sample sizes. The conclusions must be drawn appropriately based on the data presented.

Reviewer #1: Yes

Reviewer #3: Yes

**********

3. Has the statistical analysis been performed appropriately and rigorously?

Reviewer #1: Yes

Reviewer #3: Yes

**********

4. Have the authors made all data underlying the findings in their manuscript fully available?

The PLOS Data policy requires authors to make all data underlying the findings described in their manuscript fully available without restriction, with rare exception (please refer to the Data Availability Statement in the manuscript PDF file). The data should be provided as part of the manuscript or its supporting information, or deposited to a public repository. For example, in addition to summary statistics, the data points behind means, medians and variance measures should be available. If there are restrictions on publicly sharing data—e.g. participant privacy or use of data from a third party—those must be specified.

Reviewer #1: Yes

Reviewer #3: Yes

**********

5. Is the manuscript presented in an intelligible fashion and written in standard English?

PLOS ONE does not copyedit accepted manuscripts, so the language in submitted articles must be clear, correct, and unambiguous. Any typographical or grammatical errors should be corrected at revision, so please note any specific errors here.

Reviewer #1: Yes

Reviewer #3: Yes

**********

6. Review Comments to the Author

Please use the space provided to explain your answers to the questions above. You may also include additional comments for the author, including concerns about dual publication, research ethics, or publication ethics. (Please upload your review as an attachment if it exceeds 20,000 characters)

Reviewer #1: Dear Editor,

The manuscript has improved well and the authors addressed the comments.

I recommend acceptance after minor revision.

Minor points:

1. Confidence intervals (95% CI) should be included in Figure 3 (e.g., by adding error bars) to improve clarity and interpretation of the results.

2. Clarify the implications of the imprecision in the midpoint approach, particularly how it may affect the timing and accuracy of incidence estimates. For example, explain whether this approach could lead to misclassification of infection timing, distortion of temporal trends, or over- or underestimation of incidence rates when the interval between tests is long.

3. Provide a more structured comparison of the strengths and limitations of the different methods.

Best regards,

Reviewer #3: (No Response)

**********

7. PLOS authors have the option to publish the peer review history of their article (what does this mean?). If published, this will include your full peer review and any attached files.

If you choose “no”, your identity will remain anonymous but your review may still be made public.

**Do you want your identity to be public for this peer review?** For information about this choice, including consent withdrawal, please see our Privacy Policy.

Reviewer #1: **Yes:** Abdulelah Al-Ganad

Reviewer #3: No

**********

[NOTE: If reviewer comments were submitted as an attachment file, they will be attached to this email and accessible via the submission site. Please log into your account, locate the manuscript record, and check for the action link "View Attachments". If this link does not appear, there are no attachment files.]

To ensure your figures meet our technical requirements, please review our figure guidelines: https://journals.plos.org/plosone/s/figures

You may also use PLOS’s free figure tool, NAAS, to help you prepare publication quality figures: https://journals.plos.org/plosone/s/figures#loc-tools-for-figure-preparation.

NAAS will assess whether your figures meet our technical requirements by comparing each figure against our figure specifications.

# Author comment: Author response to Decision Letter 2

**DOI:** 10.1371/journal.pone.0335115.r004

20 May 2026

Round 2 Response to Reviewers

Reviewer #1: Dear Editor,

The manuscript has improved well and the authors addressed the comments.

I recommend acceptance after minor revision.

Minor points:

1. Confidence intervals (95% CI) should be included in Figure 3 (e.g., by adding error bars) to improve clarity and interpretation of the results.

Figure 3 now contains error bars representing the 95% confidence interval of each annual estimate, and this is reflected in the figure description. Point markers (e.g., circle, square, triangle) were removed for better visualization of the confidence interval bars.

2. Clarify the implications of the imprecision in the midpoint approach, particularly how it may affect the timing and accuracy of incidence estimates. For example, explain whether this approach could lead to misclassification of infection timing, distortion of temporal trends, or over- or underestimation of incidence rates when the interval between tests is long.

These points are now discussed in paragraph 6 of the discussion section:

“The test-negative method had the advantage of being able to assign the date of HCV infection to be the midpoint between HCV- and HCV+ test dates. This method has been used in previous studies to improve precision in the classification of HCV infection date by assuming that the average date of infection occurred halfway between negative and positive HCV test dates.[11, 24] While the true date of infection must fall in this window, choosing the midpoint to represent infection date risks misclassification of HCV infection date, especially when the duration between the negative and positive test is long (average of 4 years in the present study). This change inflated HCV incidence estimates earlier in follow-up and diminished them later in follow-up (Fig 5). This is because cases were only reassigned from later years to earlier years; 1999 gained HCV cases while losing none, and the ratio of lost cases to gained cases increased for each year of follow-up until 2018 when HCV cases were only lost. It is therefore important to recognize that when HCV testing data is only available for a specific window of time, reassigning infection dates to the midpoint between an HCV- and HCV+ test could lead to the potentially false conclusion that the incidence of HCV is decreasing.”

3. Provide a more structured comparison of the strengths and limitations of the different methods.

In the previous submission, the performance of each method was discussed systematically one at a time, without mention of the other methods. In the revised submission, listed strengths and limitations for a given method now also include discussion of how the other methods fare with respect to the strength or limitation in question. This gives structure to the comparison of methods without reorganizing the flow of information in the discussion section.

Attachment Submitted filename: Response to Reviewers.docx

# Editor report: Decision Letter 2

**DOI:** 10.1371/journal.pone.0335115.r005

**Authors:** Hamid Sharifi

24 May 2026

Four methods for estimating hepatitis C incidence using extant testing data

PONE-D-25-54063R2

Dear Dr. McFarlane,

We’re pleased to inform you that your manuscript has been judged scientifically suitable for publication and will be formally accepted for publication once it meets all outstanding technical requirements.

Within one week, you’ll receive an e-mail detailing the required amendments. When these have been addressed, you’ll receive a formal acceptance letter and your manuscript will be scheduled for publication.

An invoice will be generated when your article is formally accepted. Please note, if your institution has a publishing partnership with PLOS and your article meets the relevant criteria, all or part of your publication costs will be covered. Please make sure your user information is up-to-date by logging into Editorial Manager at Editorial Manager® and clicking the ‘Update My Information' link at the top of the page. For questions related to billing, please contact billing support.

If your institution or institutions have a press office, please notify them about your upcoming paper to help maximize its impact. If they’ll be preparing press materials, please inform our press team as soon as possible -- no later than 48 hours after receiving the formal acceptance. Your manuscript will remain under strict press embargo until 2 pm Eastern Time on the date of publication. For more information, please contact onepress@plos.org.

Kind regards,

Hamid Sharifi, Professor

Academic Editor

PLOS One

Additional Editor Comments (optional):

Reviewers' comments:

# Editor report: Acceptance letter

**DOI:** 10.1371/journal.pone.0335115.r006

**Authors:** Hamid Sharifi

PONE-D-25-54063R2

PLOS One

Dear Dr. McFarlane,

I'm pleased to inform you that your manuscript has been deemed suitable for publication in PLOS One. Congratulations! Your manuscript is now being handed over to our production team.

At this stage, our production department will prepare your paper for publication. This includes ensuring the following:

* All references, tables, and figures are properly cited

* All relevant supporting information is included in the manuscript submission,

* There are no issues that prevent the paper from being properly typeset

You will receive further instructions from the production team, including instructions on how to review your proof when it is ready. Please keep in mind that we are working through a large volume of accepted articles, so please give us a few days to review your paper and let you know the next and final steps.

Lastly, if your institution or institutions have a press office, please let them know about your upcoming paper now to help maximize its impact. If they'll be preparing press materials, please inform our press team within the next 48 hours. Your manuscript will remain under strict press embargo until 2 pm Eastern Time on the date of publication. For more information, please contact onepress@plos.org.

You will receive an invoice from PLOS for your publication fee after your manuscript has reached the completed accept phase. If you receive an email requesting payment before acceptance or for any other service, this may be a phishing scheme. Learn how to identify phishing emails and protect your accounts at https://explore.plos.org/phishing.

If we can help with anything else, please email us at customercare@plos.org.

Thank you for submitting your work to PLOS ONE and supporting open access.

Kind regards,

PLOS ONE Editorial Office Staff

on behalf of

Dr. Hamid Sharifi

Academic Editor

PLOS One
